# Supplementary material for: LINC00842 inactivates transcription co-regulator PGC-1α to promote pancreatic cancer malignancy through metabolic remodelling
Source: Nat Commun. 2021 Jun 22;12:3830. doi: 10.1038/s41467-021-23904-4 (PMC8219694; doi:10.1038/s41467-021-23904-4)
Supplement: Supplementary file 1 — Supplementary Information [file 41467_2021_23904_MOESM1_ESM.pdf]

Huang et al_Supplementary Figure 1

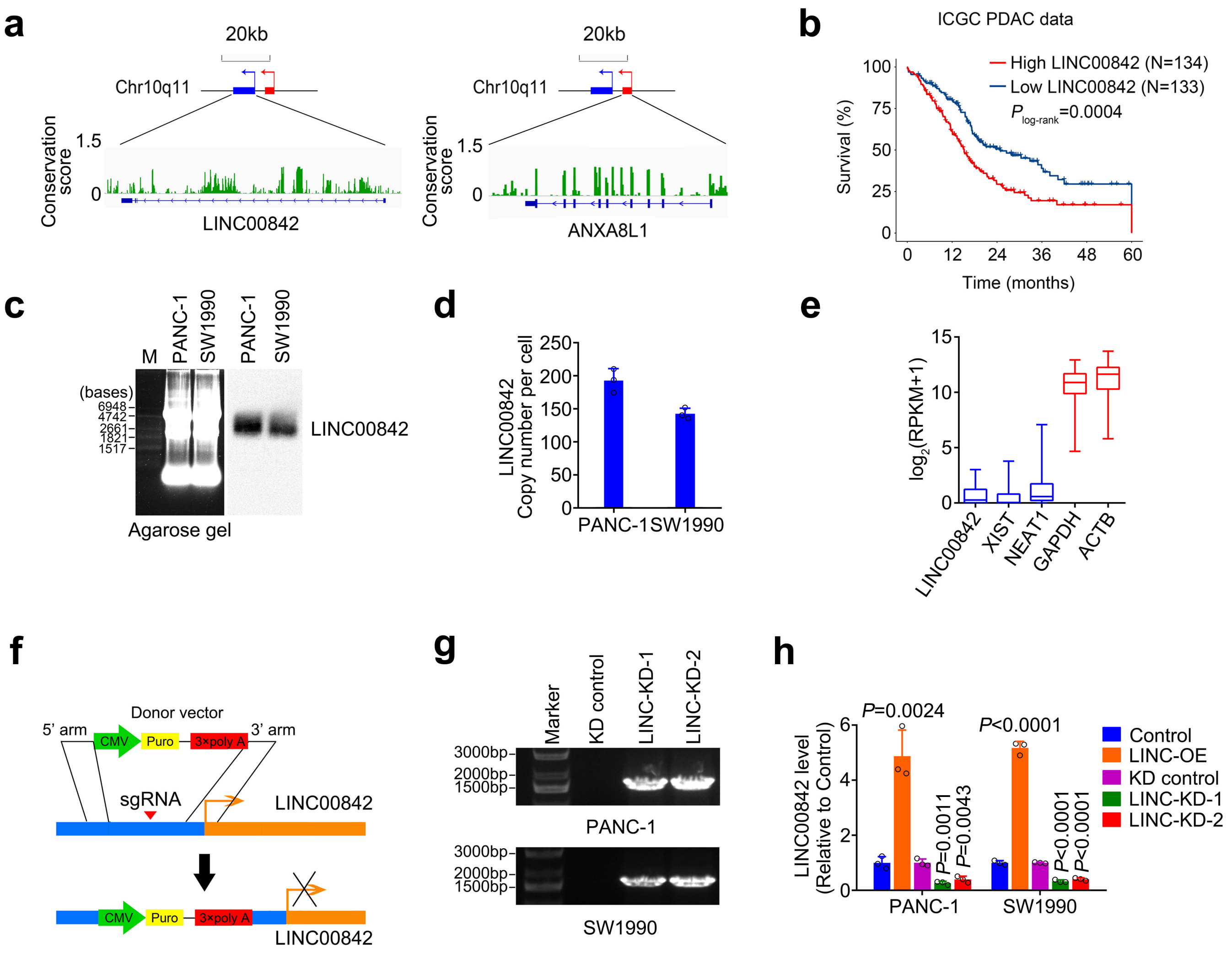

Supplementary Figure 1. Characterization of *LINC00842* and generation of PDAC cells with *LINC00842* silence.

(**a**) *LINC00842* is moderately conserved among placental mammals compared with its nearby highly conserved coding gene *ANXA8L1*. Placental mammal conservation of *LINC00842* and *ANXA8L1* by PhastCons, UCSC (http://genome.ucsc.edu/). (**b**) Kaplan-Meier estimates of survival time of PDAC patients in ICGC Cohort by different *LINC00842* levels. (**c**) Northern blot analysis of *LINC00842* in PDAC cells, showing its expected molecular size. (**d**) *LINC00842* copy numbers per PDAC cells determined by qRT–PCR. Data represent mean ± SD from 3 independent measures. (**e**) Results of in silico analysis of RNC-seq and Ribo-seq data (n = 347) indicating low affinity of *LINC00842* and other classic lncRNAs such as *XIST* and *NEAT1* to ribosome compared with classic protein coding RNAs *GAPDH* and *ACTB*. Data are shown in min to max boxplot; the lines in the middle of the box are medians and the upper and lower lines indicate 25th and 75th percentiles. (**f**) Schematic of *LINC00842* knockdown approach. A 3 × poly (A) stop cassette was knocked into the *LINC00842* promoter region via the CRISPR/Cas9 system. (**g**) Genetic integration of 3 × poly (A) insertion in PDAC cells. Cells derived from two clones of each cell line were PCR checked and designated LINC00842-KD-1 and LINC00842-KD-2. (**h**) Relative *LINC00842* levels in PDAC cells with *LINC00842* overexpression (LINC-OE) or silenced (LINC-KD). Results are mean ± SD from 3 independent determinations. The *P* values were determined by Student's *t*-test (two-tailed).

Huang et al_Supplementary Figure 2

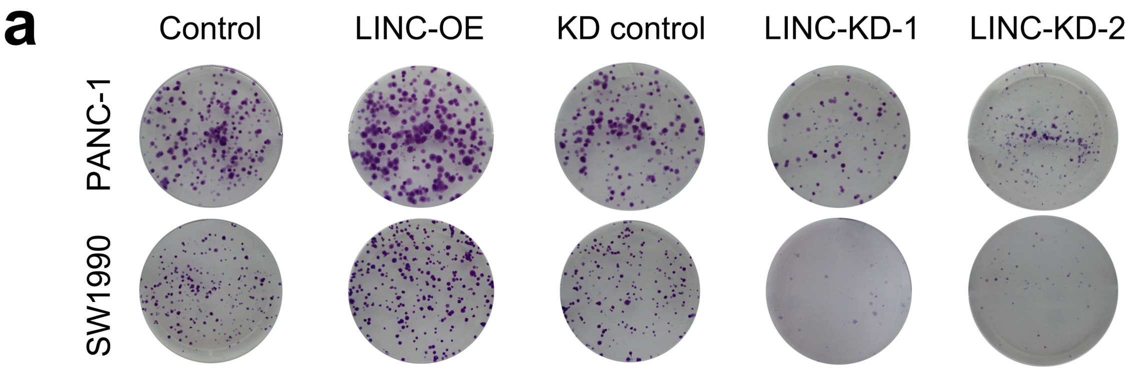

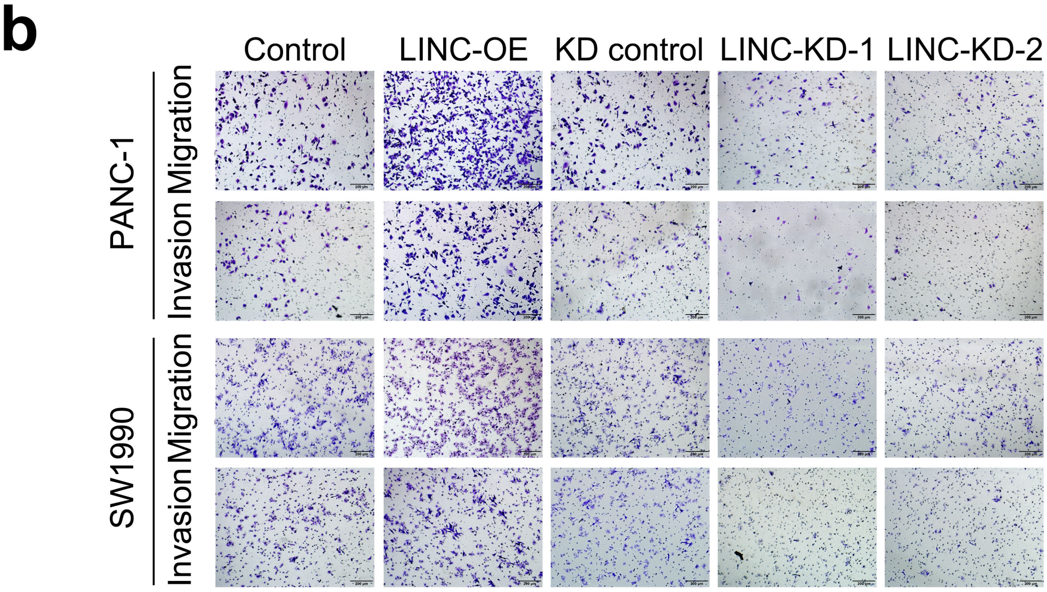

c

Metastasis of xenografts derived from PANC-1 cells

Mouse	Control group	LINC-OE group	KD control group	LINC-KD-1 group	LINC-KD-2 group
#1	No metastasis	Lung, Liver, Mesentery	No metastasis	No metastasis	No metastasis
#2	No metastasis	Liver, Mesentery	No metastasis	No metastasis	No metastasis
#3	No metastasis	Lung, Liver, Mesentery	No metastasis	No metastasis	Mesentery
#4	Liver	Mesentery	No metastasis	No metastasis	No metastasis
#5	Mesentery	Lung, Liver, Mesentery	Mesentery	No metastasis	No metastasis
#6	Liver, Mesentery	Liver, Mesentery	Liver	No metastasis	No metastasis
#7	Liver	Lung, Mesentery	Mesentery	No metastasis	No metastasis
#8	Mesentery	Lung, Liver, Mesentery	Liver, Mesentery	No metastasis	No metastasis

Metastasis of xenografts derived from SW1990 cells

Mouse	Control group	LINC-OE group	KD control group	LINC-KD-1 group	LINC-KD-2 group
#1	No metastasis	Lung, Liver, Mesentery	No metastasis	No metastasis	No metastasis
#2	No metastasis	Mesentery	No metastasis	Liver	No metastasis
#3	No metastasis	Liver, Mesentery	No metastasis	No metastasis	No metastasis
#4	Mesentery	Lung, Liver, Mesentery	Mesentery	No metastasis	No metastasis
#5	Lung, Mesentery	Liver, Mesentery	Mesentery	No metastasis	No metastasis
#6	Liver	Lung, Mesentery	Lung, Liver	No metastasis	Mesentery
#7	Mesentery	Lung, Liver, Mesentery	Mesentery	No metastasis	No metastasis
#8	Mesentery	Liver, Mesentery	Liver	No metastasis	No metastasis

Supplementary Figure 2. *LINC00842* promotes malignant phenotypes of PDAC cells.

(**a** and **b**) Representative pictures showing the effect of *LINC00842* overexpression (LINC-OE) or silence (LINC-KD) on colony formation (**a**) and migration and invasion (**b**) of PDAC cells. Scale bars in (**b**), 200 µm. Pictures are representative results from 3 random fields with similar results. (**c**) Organs with metastatic tumors from implanted PDAC xenograft in pancreas in each mouse. Quantified data are shown in Figure 1.

Huang_et al Supplementary Figure 3

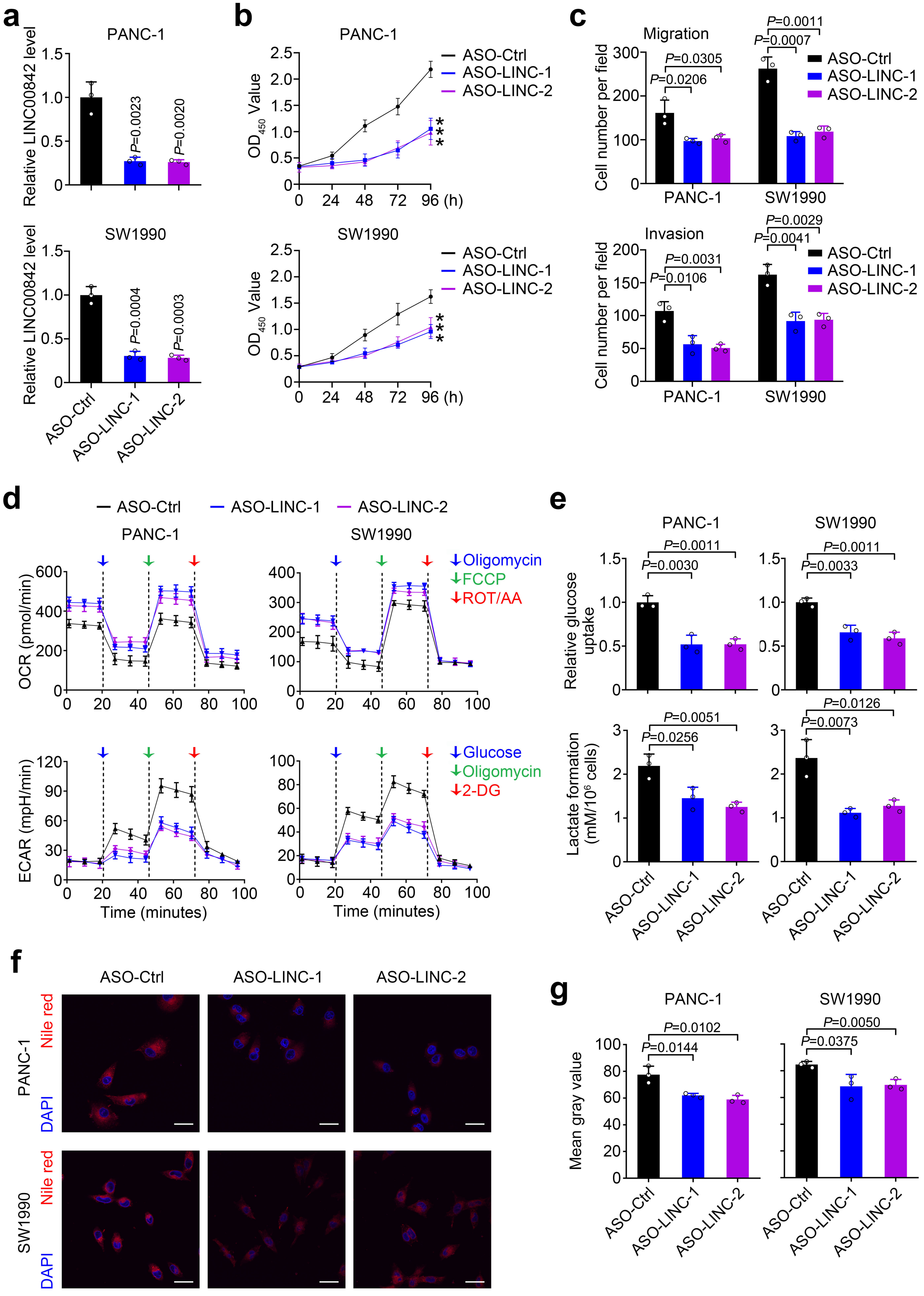

Supplementary Figure 3. Knockdown of *LINC00842* by ASO prevents malignant phenotypes of PDAC cells.

(**a**) *LINC00842* levels in PDAC cells transfected with antisense oligonucleotide *LINC00842* (ASO-LINC) or ASO control. (**b** and **c**) Effects of *LINC00842* knockdown (ASO-LINC-1 and ASO-LINC-2) on abilities of PDAC cell proliferation (**b**, n = 4), migration and invasion (**c,** Data are mean ± SD from 3 random fields). (**d**) Effects of *LINC00842* silence (ASO-LINC-1 and ASO-LINC-2) on oxygen consumption rate (OCR) (*upper*) or extracellular acidification rate (ECAR) (*lower*). (**e**) Effects of *LINC00842* depletion on glucose uptake (*upper*) or lactate production (*lower*). (**f** and **g**) Representative pictures of Nile red (red) and DAPI (blue) staining of cells with *LINC00842* silencing (**f**) and quantification using Image J software (**g**). Scale bar, 30 µm. The results in (**a**), (**b**), (**d**), (**e**) and (**g**) are mean ± SD from 3 independent experiments. The *P* values of (**a**), (**c**), (**e**), (**g**) and ***, *P* < 0.001 in (**b**) were determined by Student's *t*-test (two-tailed).

Huang et al_Supplementary Figure 4

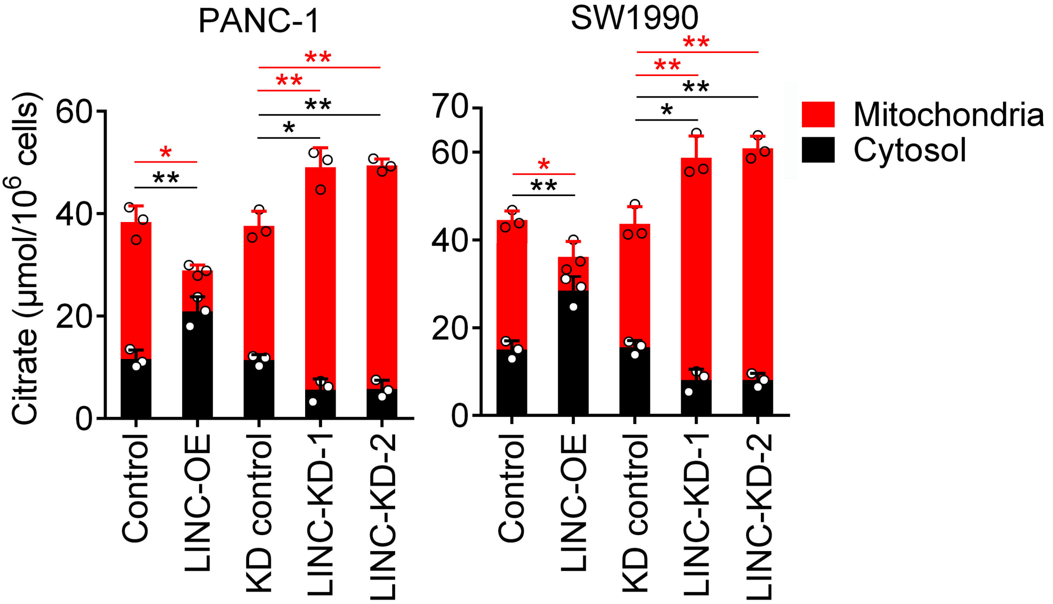

Supplementary Figure 4. Effect of *LINC00842* overexpression (LINC-OE) or silence (LINC-KD) on citrate distribution in the mitochondria and cytosol of cells. Black colour indicates cytosol and red colour indicates mitochondria. Results are mean ± SD from 3 independent experiments. *, $P < 0.05$ and **, $P < 0.01$ of Student's *t*-test (two-tailed. No adjustments were made for multiple comparisons).

Huang et al_Supplementary Figure 5

a

>LINC00842 1-690nt
GCGGGGGCGGGCGCGCGGGGCCUUGCCGGAGAACCUGACUCUCCGCAGCA
GCAGUGGAAGCCGGAGUGACGCGUUGUGUUGAACACCAGUUUUCUGGAGCG
CUGUGUGUUCUUAACAGCUGAGCAGUCUGUUUCUCCAAUCAGGUUUCAAAG
CCACUUCAACUGCACUGGCCCCUGUGGGUCACUGCUGCACCGCCCUGACCC
AUGUGGGUCCCUGAGGAGCGACCUGCCGGGGCCACCUGGCUGGACGAAAAA
GACACACCUUGGACUUAAGCCGUGAGAAAAAAACUUCAUCAGUAAGAAGAAU
GAAUAAACAGACUAGGUUGAAUCCAUACAAUGGAAUGUUAGCAGACAAUAAA
AAGAAAAUGAACUAUUGAUGUCCCCUACUGCACAGCAGAAGCUCUGAAUCGU
GUUCCUGAAUGAAAGAAGUCAGAGAUGAAAAGAUGGGCCAGGAGUCCAGUU
UCUGGAAGGCCAAGAAUCGAAGUAGCAAGCUGCAGCCGUUUCCAGACAAG
CAGGAUGUGGGGAUGCAGAAGAAUUCAGGACUGGAGGGGCAAACUCCGAUG
UGACUGAGGCCCCACUGCCAAAUGGCAGCAUGCUCAGAUAGCACCCAGGAAU
UUGGGGAAAAAACUGGUGCUCACAGCUGCCCAGUUAAGGCACAAGUCUCCC
GCCUGCUGCAGGGGUUGUGAGGU

WT	MUT
Position 1: …GGUCACUGCUGCACC…	Position 1: …UUGACAGUAGUACAA…
Position 2: …GGUCCCUGAGGAGCGACC…	Position 2: …UUGAAAGUCUUCUAUCAA…
Position 3: …UGAACUAUUGAUGUCCC…	Position 3: …GUCCAGCGGUCGUGAAA…
Position 4: …UCUGAAUCGUG…	Position 4: …GAGUCCGAUGU…
Position 5: …UGUGACUGAGGCCCCA…	Position 5: …GUGUCAGUCUUAAAAC…
Position 6: …UAGCACCCAGGAAUUU…	Position 6: …GCUACAAACUUCCGGG…

b

RNA pull-down

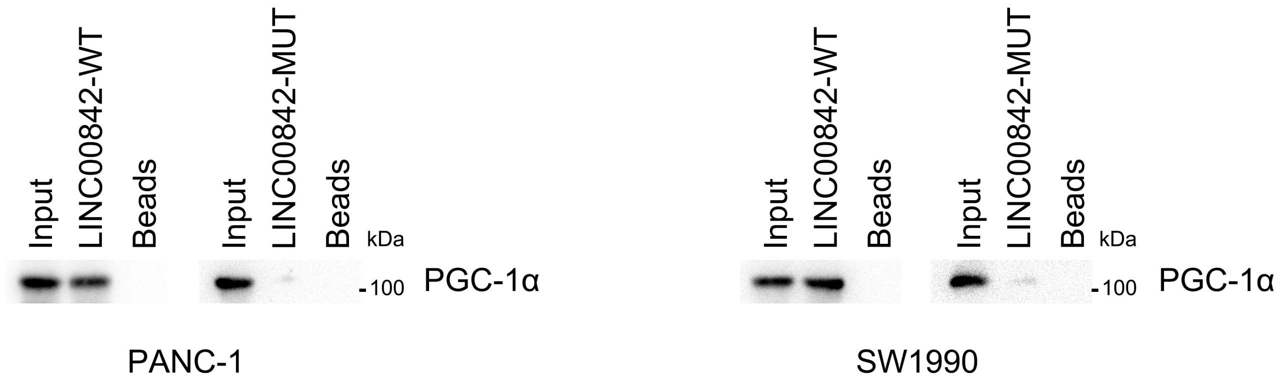

Supplementary Figure 5. Identification of binding sites within 5'-end of *LINC00842*.

(**a**) Prediction binding sites of 5'-end of *LINC00842* using PRIdictor and followed by RPISeq, six positions (prediction using RF (Random Forest) classifier > 0.5) were considered for further validation (*upper*), the wild type and mutant sequences for each position were showed in *lower* panel. (**b**) Immunoblot analysis of PGC-1α retrieved by *in vitro* transcribed biotinylated wild type or mutant *LINC00842*. The immunoblot is representative result from 3 independent experiments with similar results.

Huang et al_Supplementary Figure 6

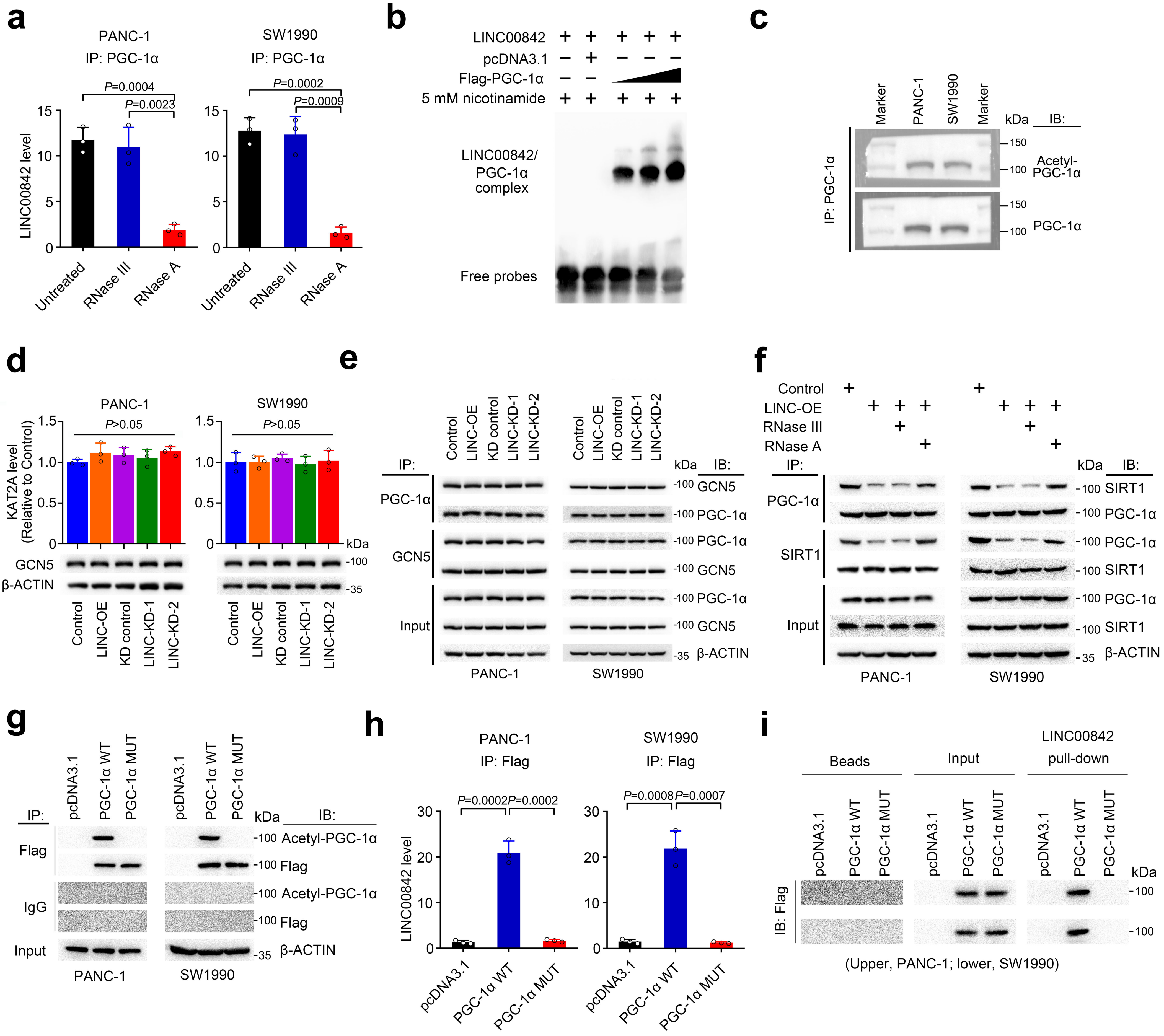

Supplementary Figure 6. Interaction pattern among *LINC00842*, PGC-1α and SIRT1 in PDAC cells. (**a**) Effect of RNase III (100 U/ml) or RNase A (10 µg/ml) treatment on the interaction of PGC-1α with *LINC00842.* The level of *LINC00842* RNA in RNA immunoprecipitation product significantly decreased in upon RNase A treatment. (**b**) RNA electrophoretic mobility shift assays of precipitated Flag-tagged PGC-1α with *LINC00842* sense strand. The probes were maintained constantly while a gradient of 0–8 µM precipitated Flag-tagged PGC-1α was added to the reactions. (**c**) Immunoprecipitation coupled with immunoblot analysis of PGC-1α and acetyl-PGC-1α in PDAC cells, showing its expected molecular size. (**d**) Effect of *LINC00842* expression change on *KAT2A* mRNA (*upper panel*) and protein (GCN5, *lower panel*) levels. (**e**) No significant effect of *LINC00842* expression change on PGC-1α and GCN5 protein interaction. (**f**) Immunoprecipitation and immunoblotting assays show the effect of RNase A treatment on PGC-1α and SIRT1 interaction when overexpressed *LINC00842*. (**g**) Immunoprecipitation and immunoblotting assays confirm mutant PGC-1α abolish its acetylation. (**h**) RIP assays show mutant PGC-1α abolish the interaction between PGC-1α and *LINC00842*. (**i**) RNA pull-down assays show *LINC00842* cannot interact with mutant PGC-1α in PDAC cells. The results in (**a**), (**d**) and (**h**) are mean ± SD from 3 independent experiments. The *P* values were determined by Student's *t*-test (two-tailed).

Huang et al_Supplementary Figure 7

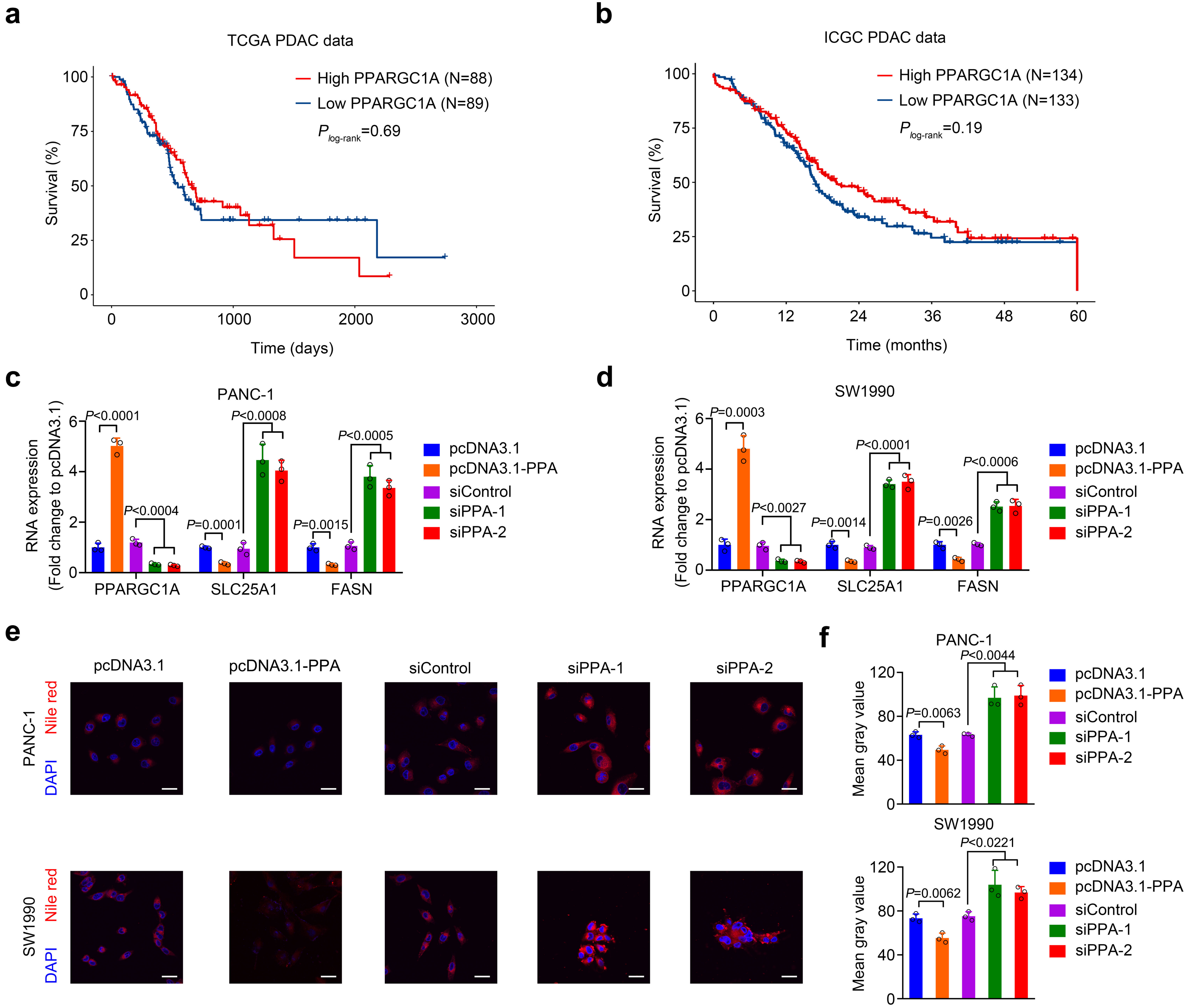

Supplementary Figure 7. PGC-1α suppresses fatty acid synthesis in PDAC cells.

(**a** and **b**) *PPARGC1A* RNA level is not associated with survival time in PDAC patients in TCGA (a) and ICGC (b) cohort. (**c** and **d**) Effects of overexpression wild type or mutant *PPARGC1A* (PPA WT and PPA MUT, respectively), and *PPARGC1A* silencing (siPPA-1 and siPPA-2) on the expression of *SLC25A1* and *FASN* in PANC-1 (**c**) and SW1990 (**d**) cells. (**e** and **f**) Representative pictures of Nile red (red) and DAPI (blue) staining of cells with overexpression wild type or mutant *PPARGC1A* (PPA WT and PPA MUT, respectively), and *PPARGC1A* silencing (siPPA-1 and siPPA-2) (**e**) and quantification using Image J software (**f**). Scale bar, 30 µm. The results in (**c**), (**d**) and (**f**) are mean ± SD from 3 independent experiments. The *P* values were determined by Student's *t*-test (two-tailed).

Huang et al_Supplementary Figure 8

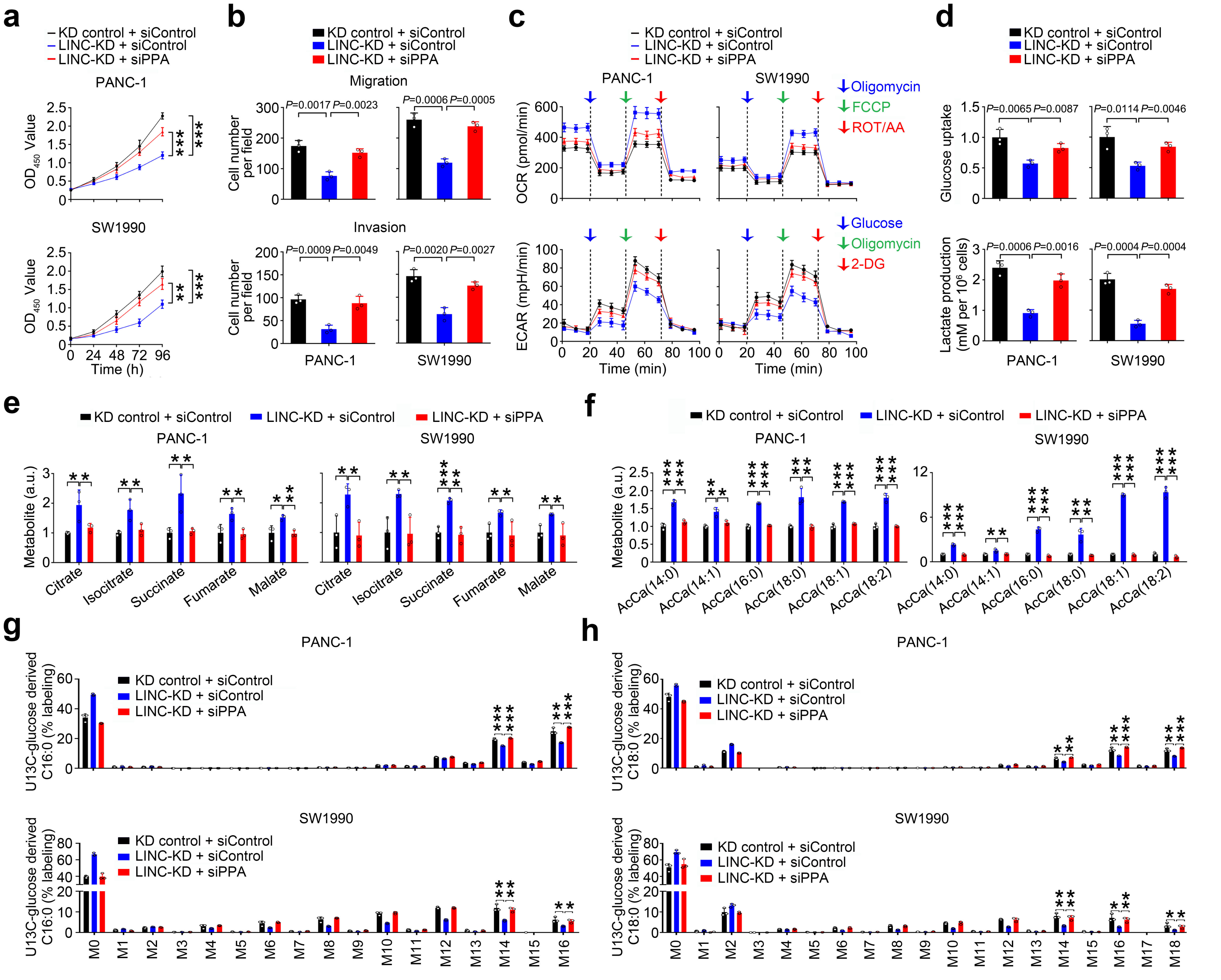

Supplementary Figure 8. *LINC00842* alters metabolic remodelling via PGC-1α in PDAC cells.

(**a**–**d**) Effects of *PPARGC1A* (PPA) depletion on malignant phenotypes (**a**, n = 4 and **b**, Data are mean ± SD from 3 random fields) and glucose metabolism (**c** and **d**, n = 3) in cells with *LINC00842* knockdown (LINC-KD). OCR, oxygen consumption rate; ECAR, extracellular acidification rate. (**e** and **f**) *PPARGC1A* depletion decreased the levels of tricarboxylic acid cycle metabolites (**e**, n = 3) and long-chain acylcarnitines (**f**, n = 3) in cells with LINC-KD. (**g** and **h**) *PPARGC1A* depletion restored palmitate (C16:0, **g**, n = 3) and stearate (C18:0, **h**, n = 3) levels in cells with LINC-KD. Results in (**a**), (**c**–**h**) represent mean ± SD from 3 independent experiments. The *P* values of (**b**), (**d**) and *, $P < 0.05$; **, $P < 0.01$ and ***, $P < 0.001$ in (**a**), (**e**–**h**) were determined by Student's *t*-test (two-tailed).

Huang et al_Supplementary Figure 9

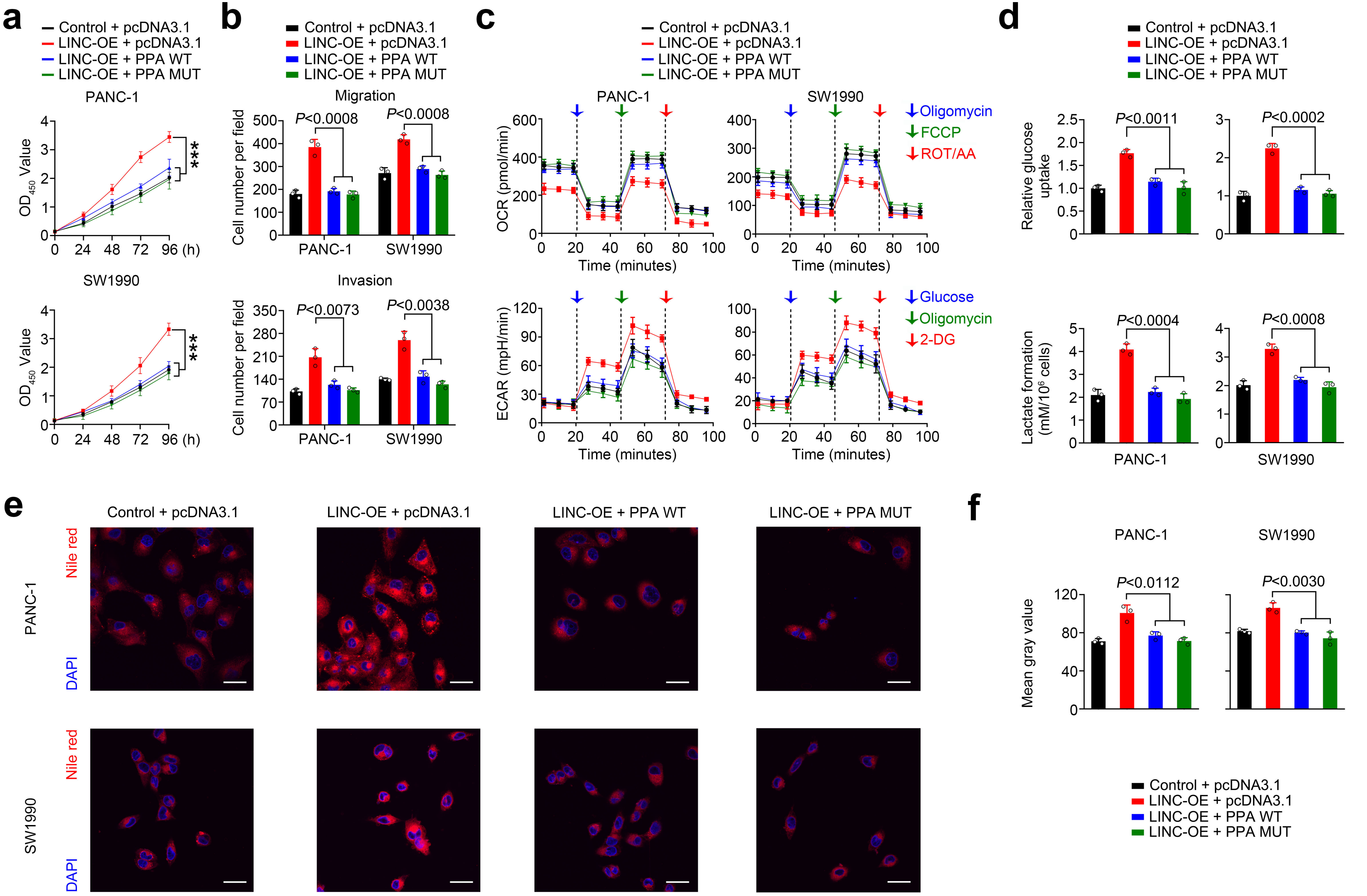

Supplementary Figure 9. *LINC00842* alters metabolic remodelling via acetylated PGC-1α in PDAC cells.

(**a**–**d**) Effects of wild type or mutant *PPARGC1A* (PPA WT and PPA MUT, respectively) overexpression on malignant phenotypes (**a**, n = 4 and **b**, Data are mean ± SD from 3 random fields) and glucose metabolism (**c** and **d**) in cells with *LINC00842* overexpression (LINC-OE). OCR, oxygen consumption rate; ECAR, extracellular acidification rate. (**e** and **f**) Representative pictures of Nile red (red) and DAPI (blue) staining of cells with overexpression wild type or mutant *PPARGC1A* (PPA WT and PPA MUT, respectively) in cells with LINC-OE (**e**) and quantification using Image J software (**f**). Scale bar, 30 µm. The results in (**a**), (**c**), (**d**), (**f**) are mean ± SD from 3 independent experiments. The *P* values of (**b**), (**d**), (**f**) and ***, $P < 0.001$ in (**a**) were determined by Student's *t*-test (two-tailed).

Huang et al_Supplementary Figure 10

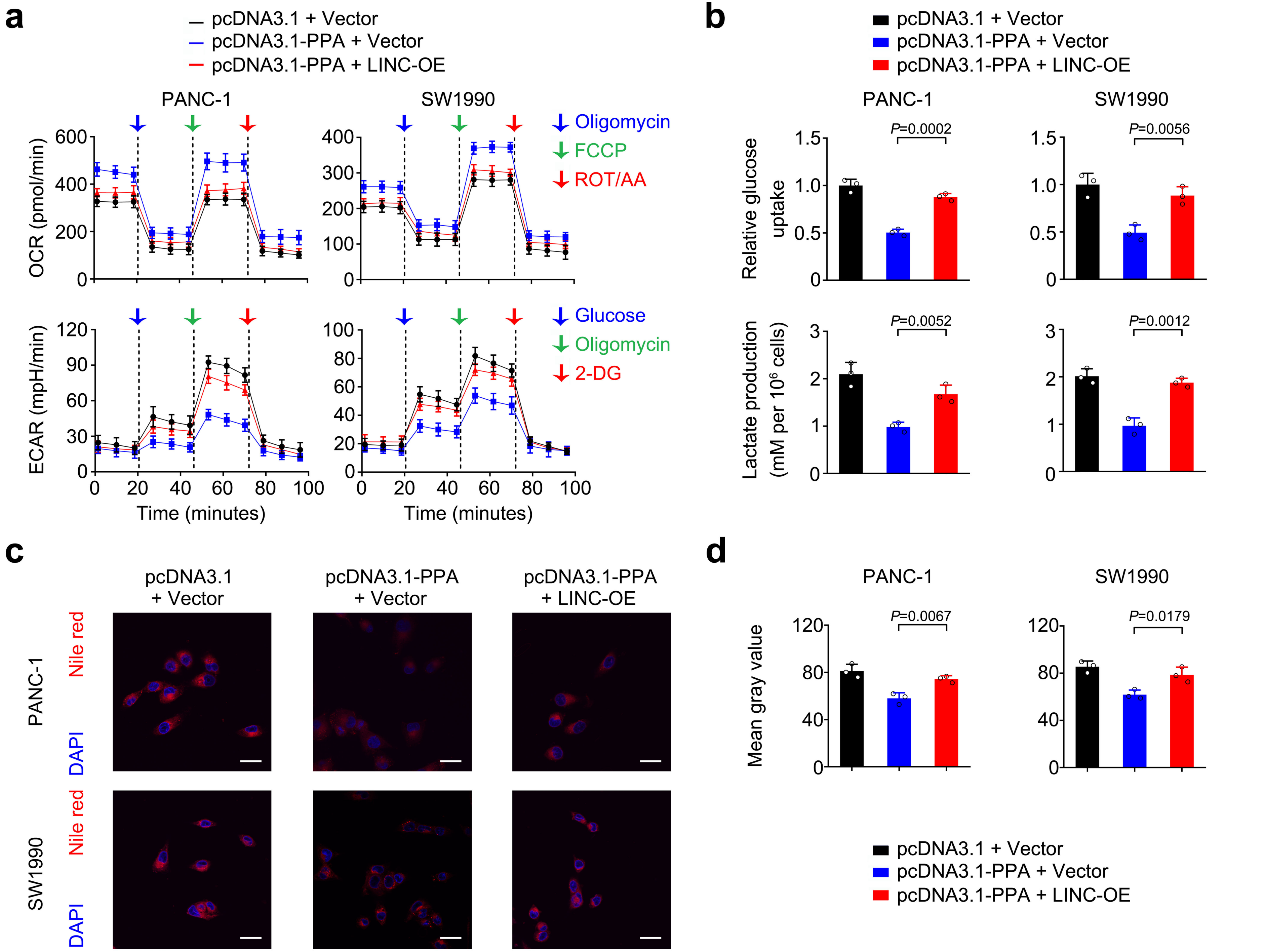

Supplementary Figure 10. *LINC00842* restores metabolic remodelling mediated by PGC-1α in PDAC cells.

(**a** and **b**) Effects of *LINC00842* overexpression (LINC-OE) on glucose metabolism in cells with *PPARGC1A* (PPA) overexpression. OCR, oxygen consumption rate; ECAR, extracellular acidification rate. (**c** and **d**) *LINC00842* overexpression restored the levels of lipid contents in cells with *PPARGC1A* (PPA) overexpression. Representative pictures of Nile red (red) and DAPI (blue) staining cells are shown (**c**) and quantification using Image J software (**d**). Scale bar, 30 µm. The results in (**a**) (**b**) and (**d**) are mean ± SD from 3 independent experiments. The *P* values of (**b**), (**d**) were determined by Student's *t*-test (two-tailed).

Huang et al_Supplementary Figure 11

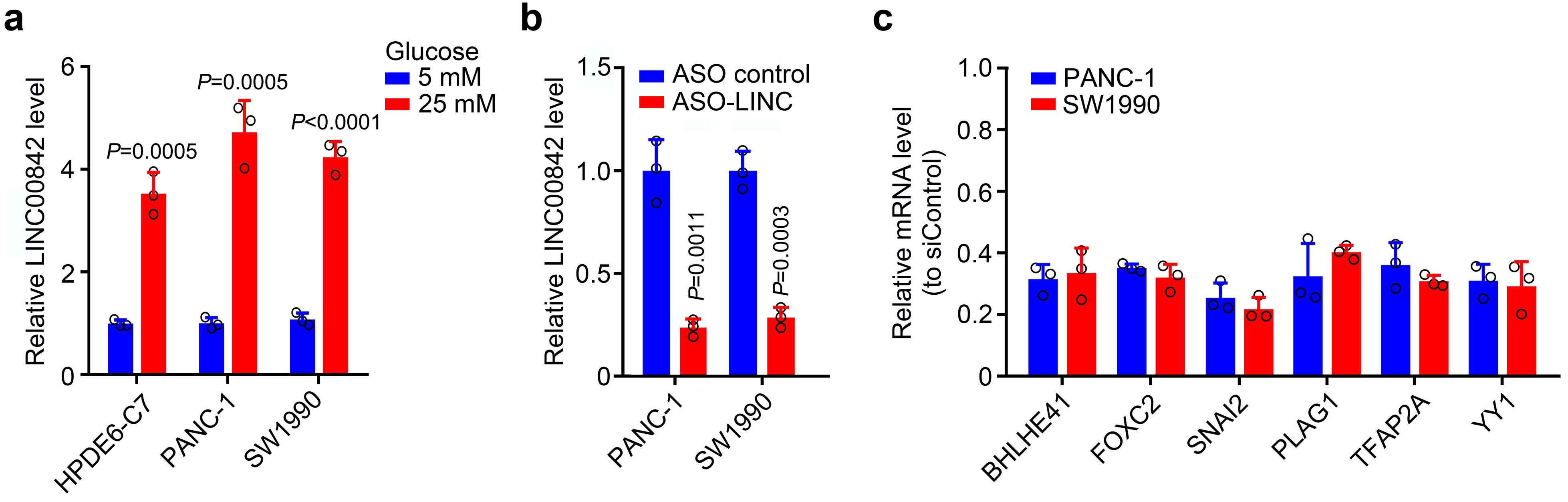

Supplementary Figure 11. High glucose promotes *LINC00842* expression via transcription factor YY1.

(**a**) *LINC00842* expression levels in immortalized human pancreatic cell HPDE6-C7 and PDAC cells cultured with 5 or 25 mM of glucose. (**b**) *LINC00842* levels in PDAC cells transfected with antisense oligonucleotide *LINC00842* (ASO-LINC) or ASO control. (**c**) siRNA silencing efficiencies of *BHLHE41*, *FOXC2*, *SNAI2*, *PLAG1*, *TFAP2A* or *YY1* expression. All results are mean ± SD relative to indicated control from 3 independent experiments. The *P* values in (**a**), (**b**) were determined by Student's *t*-test (two-tailed).

Huang et al_Supplementary Figure 12

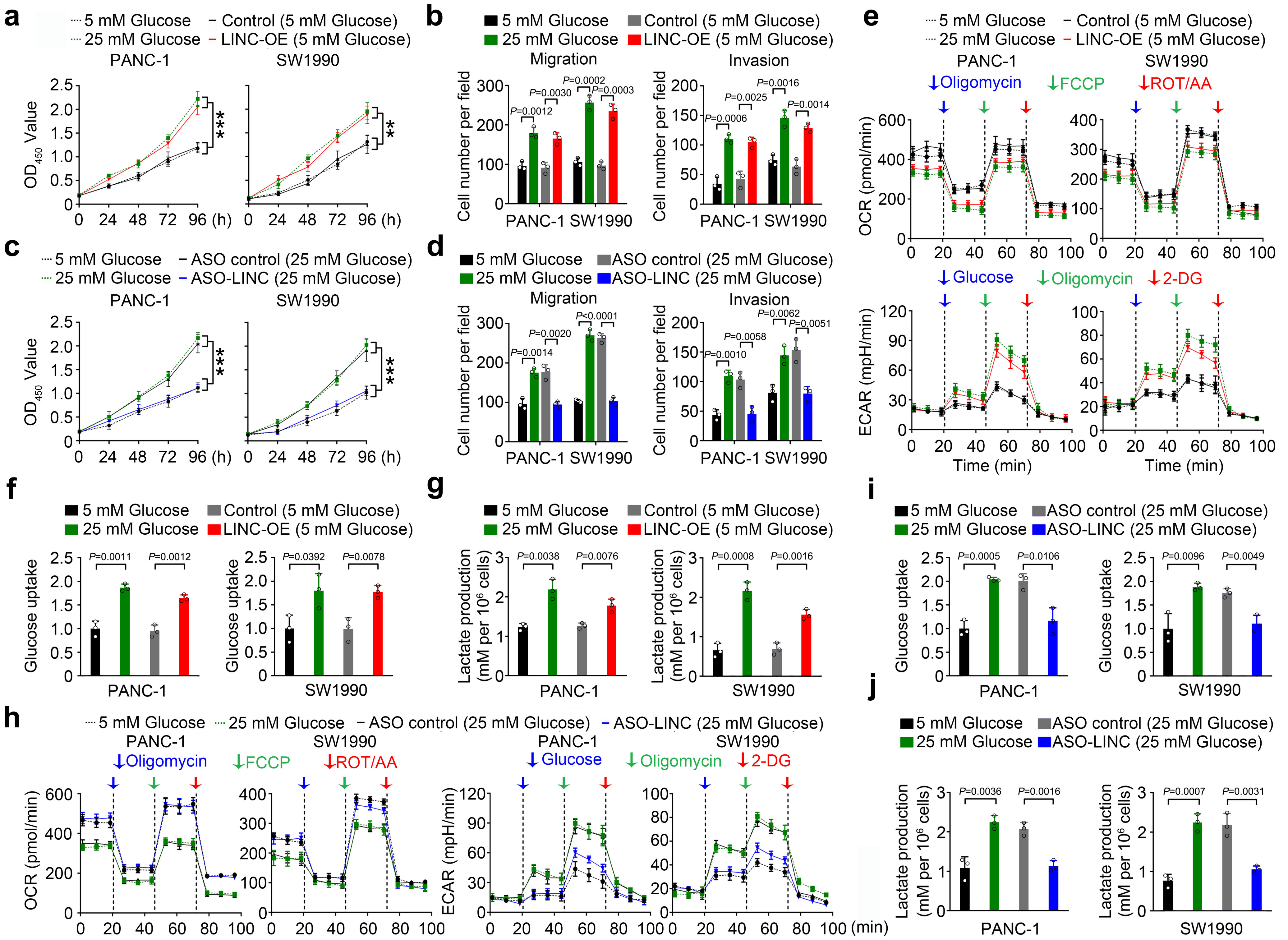

Supplementary Figure 12. Effects of *LINC00842* expression change on malignant phenotypes of PDAC cells cultured with high or low dose of glucose.

(**a** and **b**) Effects of *LINC00842* overexpression (LINC-OE) on proliferation (**a**, n = 4) and migration and invasion (**b**, Data are mean ± SD from 3 random fields) of cells exposed to 5 mM of glucose in culture. (**c** and **d**) Effects of antisense oligonucleotide *LINC00842* (ASO-LINC) on proliferation (**c**, n = 4) and migration and invasion (**d**, Data are mean ± SD from 3 random fields) of cells exposed to 25 mM of glucose in culture. (**e**–**g**) Effects of *LINC00842* overexpression on glucose metabolism in cells exposed to 5 mM of glucose in culture. (**h**–**j**) Effects of ASO-LINC on glucose metabolism in cells exposed to 25 mM of glucose in culture. OCR, oxygen consumption rate; ECAR, extracellular acidification rate. Results in (**a**), (**c**), (**e**–**j**) represent mean ± SD from 3 independent experiments. The P values of (**b**), (**d**), (**f**), (**g**), (**i**), (**j**) and **, $P < 0.01$; ***, $P < 0.001$ in (**a**), (**c**) were determined by Student's t test (two-tailed).

Huang et al_Supplementary Figure 13

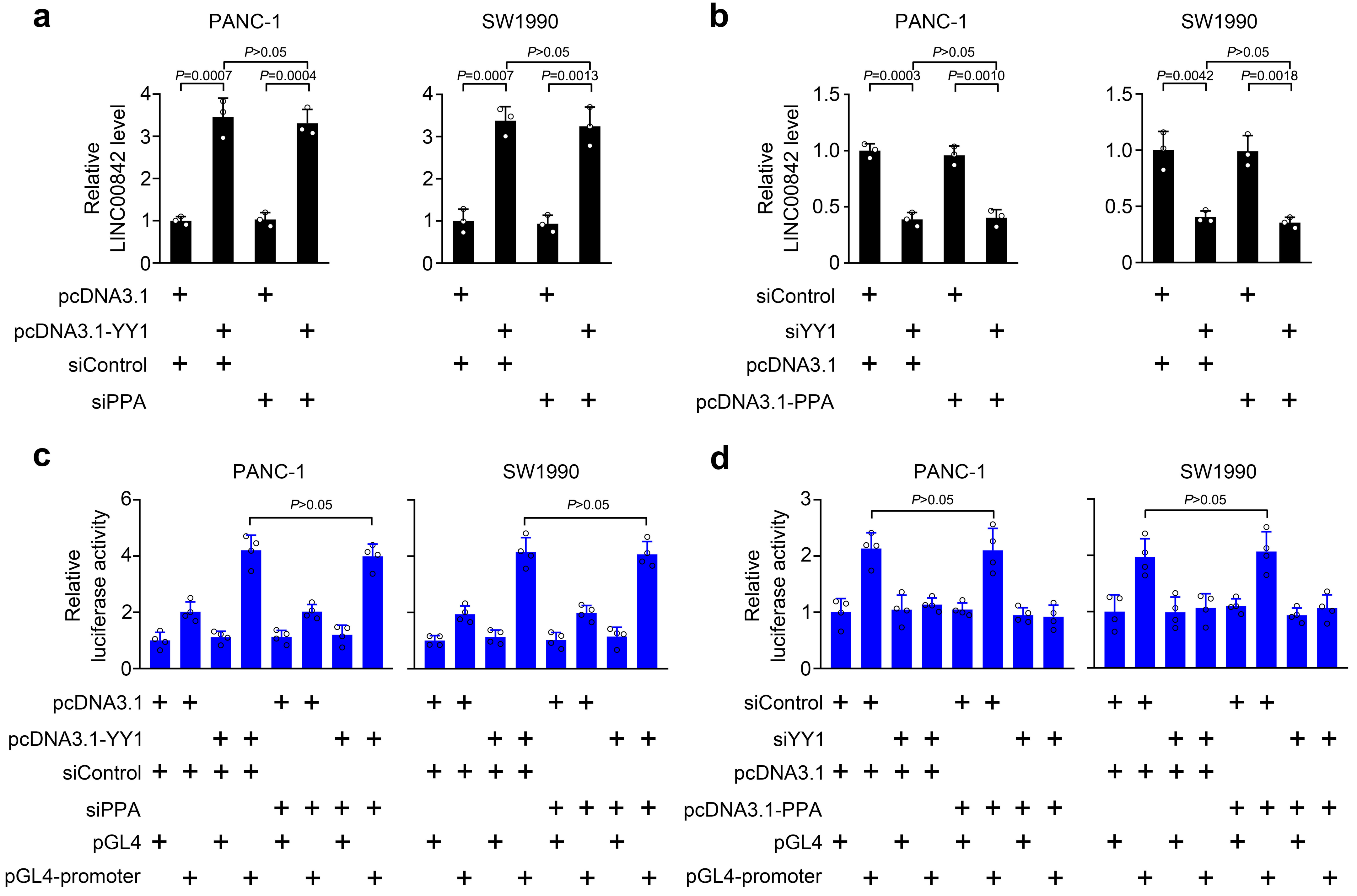

Supplementary Figure 13. PGC-1α has no effect on YY1 mediate *LINC00842* expression.

(**a**) Effects of *PPARGC1A* (PPA) depletion on *LINC00842* expression in cells with *YY1* overexpression. (**b**) Effects of *PPARGC1A* (PPA) overexpression on *LINC00842* expression in cells with *YY1* depletion. (**c** and **d**) Luciferase reporter assays in cells co-transfected with the indicated plasmids or siRNA for 48 h. All results are mean ± SD from 3 independent experiments. The *P* values in (**a**–**d**) were determined by Student's *t*-test (two-tailed).

Huang et al_Supplementary Figure 14

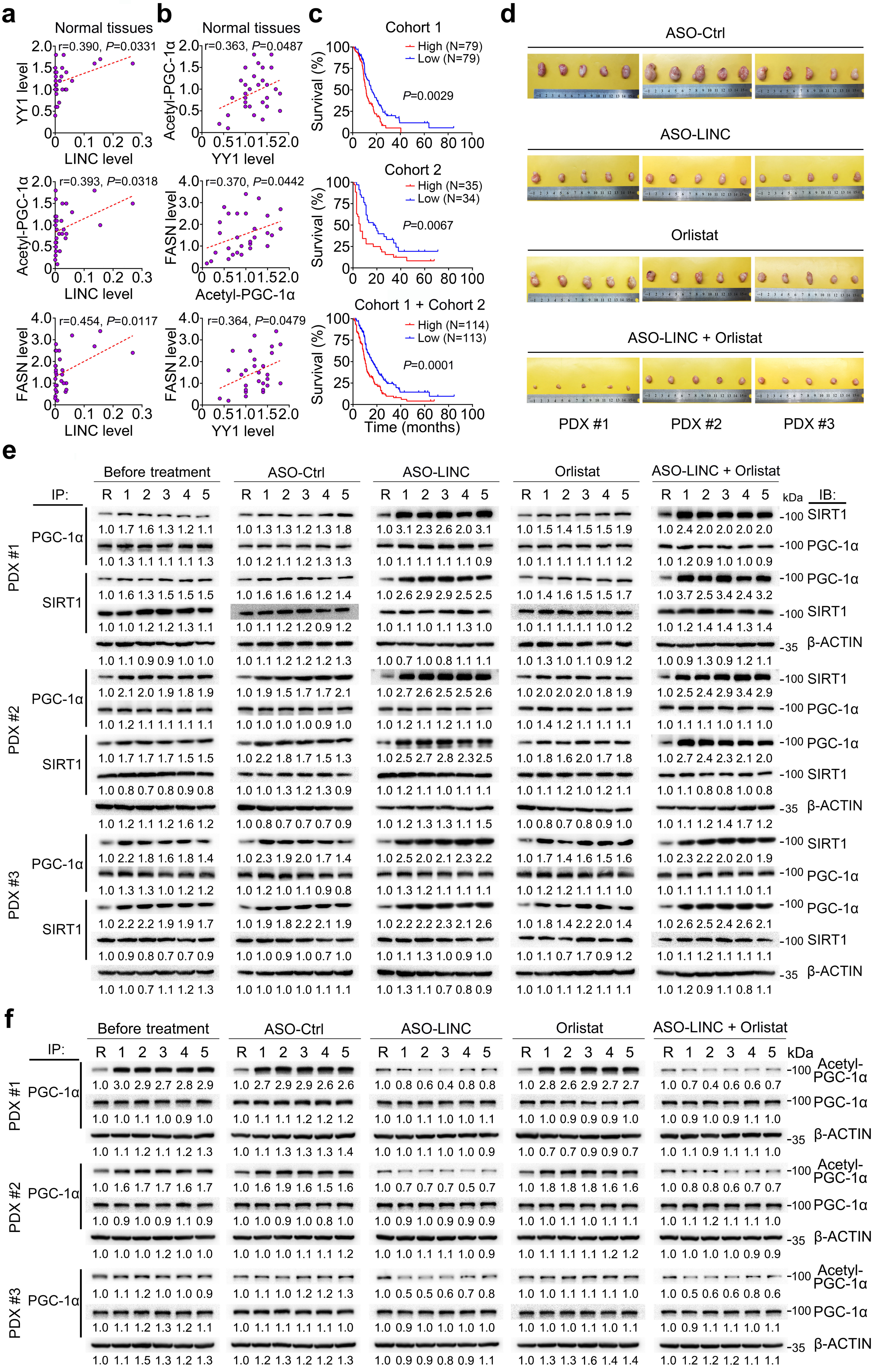

Supplementary Figure 14. *LINC00842* is a potential therapeutic target for PDAC.

(**a** and **b**) Spearman's correlations between levels of *LINC00842* (LINC) and YY1, acetyl-PGC-1α or FASN in normal pancreatic tissues (n = 30) indicated in **Fig. 7a**. (**c**) Kaplan-Meier estimates of patient's survival time in Cohort 1, Cohort 2 and pooled sample by *FASN* RNA levels in PDAC. The median *FASN* RNA levels in Cohort 1 and Cohort 2 were 0.3157 and 0.1664, respectively. High or low *FASN* level was defined by > median or ≤ median (log-rank test, two-sided. No adjustments were made for multiple comparisons). (**d**) Tumors of mice with patient-derived xenograft (PDX) treated with antisense oligonucleotide (ASO)-control (Ctrl), ASO-LINC00842, Orlistat or combination of ASO-LINC00842 and Orlistat. (**e**) The interaction between PGC-1α and SIRT1 in PDX before or after treatment with ASO-LINC00842, Orlistat or combination of ASO-LINC00842 and Orlistat (**f**) Acetyl-PPA level in mouse PDX before or after treatment with ASO-LINC00842, Orlistat or combination of ASO-LINC00842 and Orlistat shown in **Fig. 7k**. Western blot bands were quantified with Image J. The immunoblots shown in (**e**) and (**f**) are the results from once experiment.

Huang et al_Supplementary Figure 15

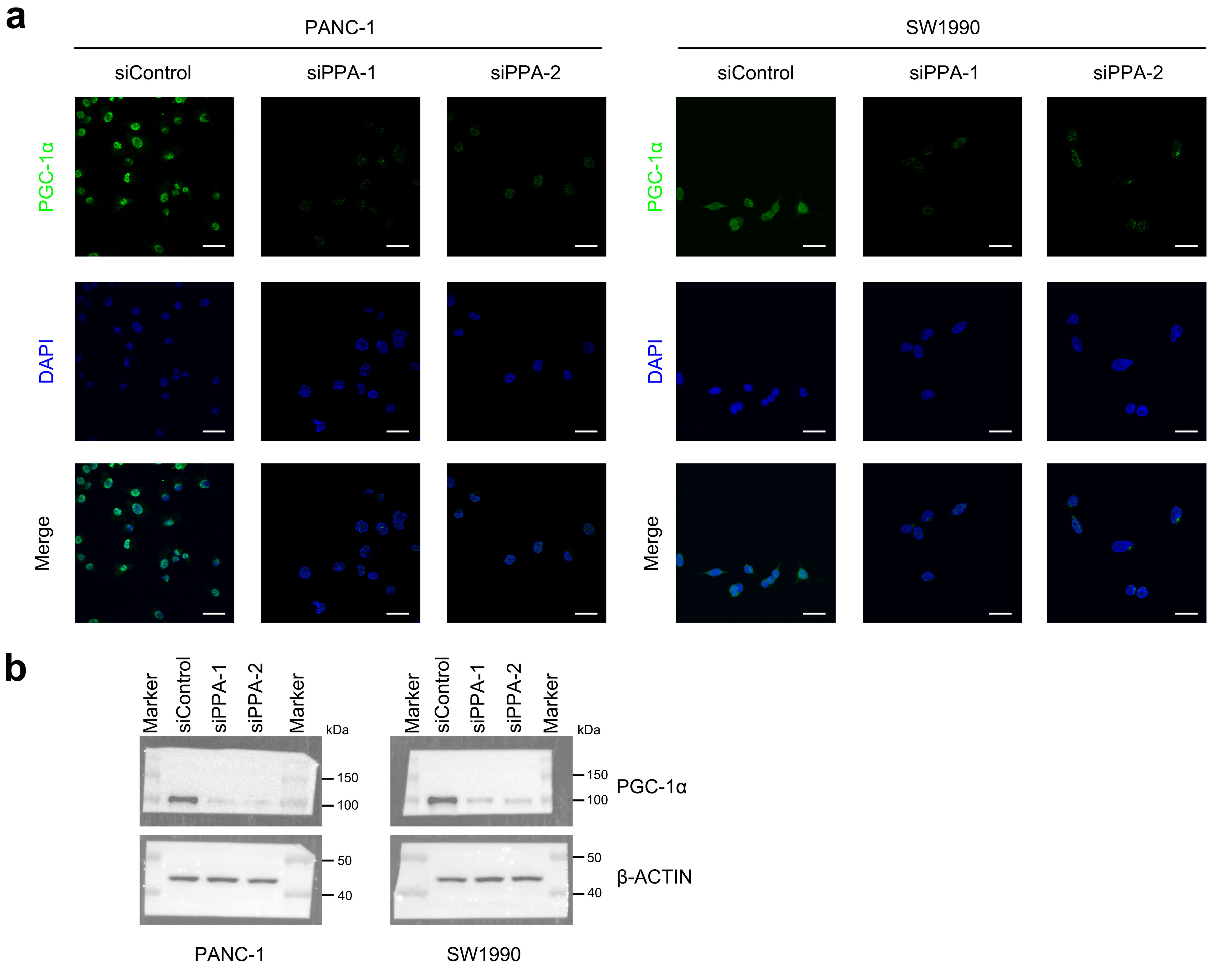

Supplementary Figure 15. Antibody validation of PGC-1α in PDAC cell lines.

(**a**) Immunofluorescence assays show knockdown of PGC-1α (green) in PANC-1 and SW1990 cell lines. Scale bar, 30 μm. (**b**) Western blot assays show knockdown of PGC-1α in PANC-1 and SW1990 cell lines. The results in (**a**) and (**b**) are representative results from 3 independent experiments with similar results.

Huang et al_Supplementary Figure 16

Figure 4c

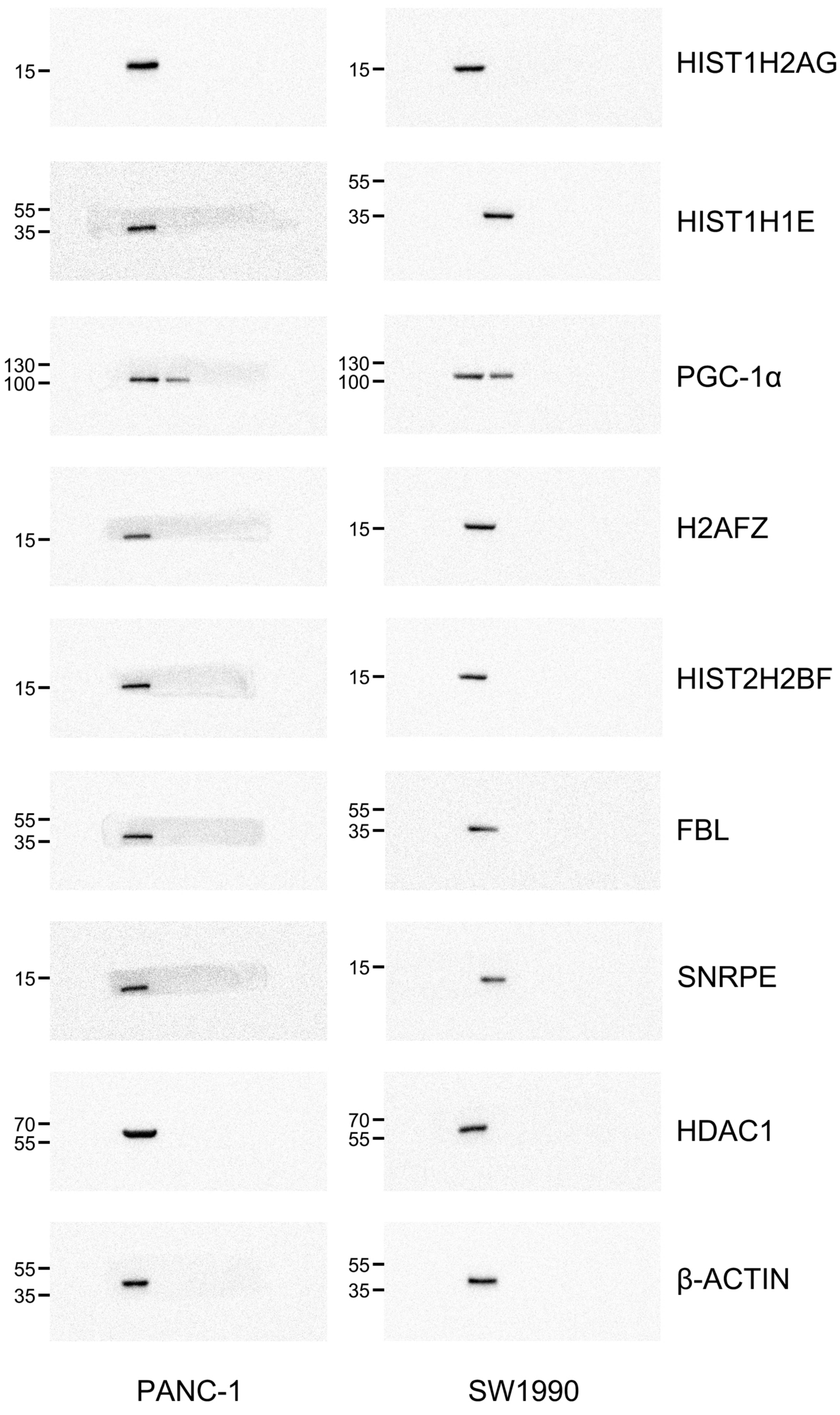

Figure 4e

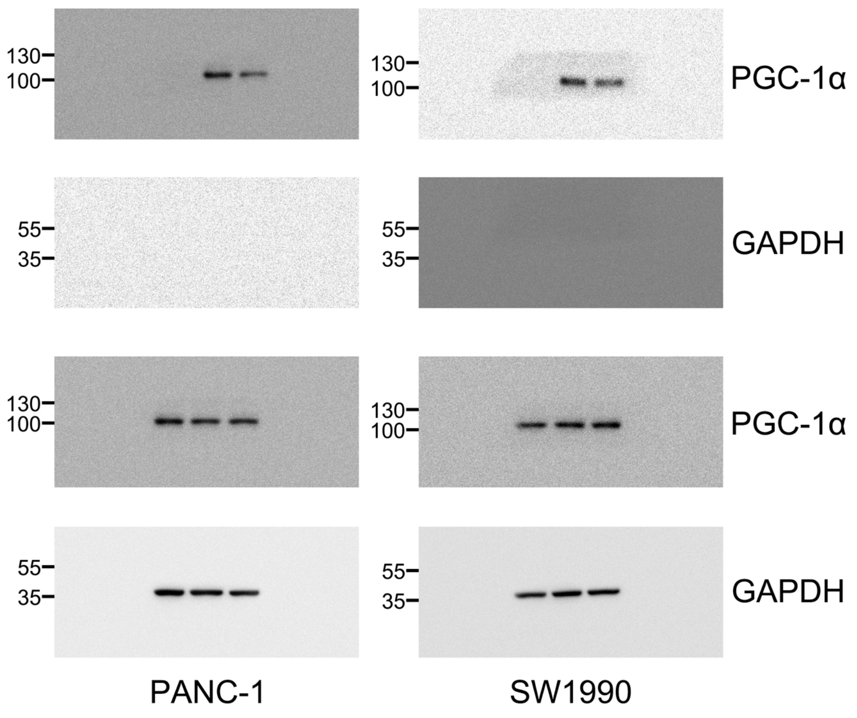

Figure 4g

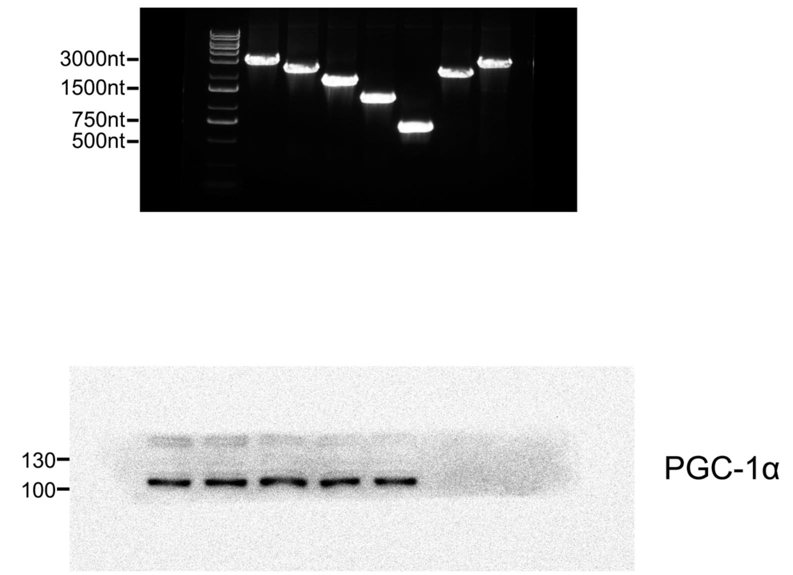

Figure 4i

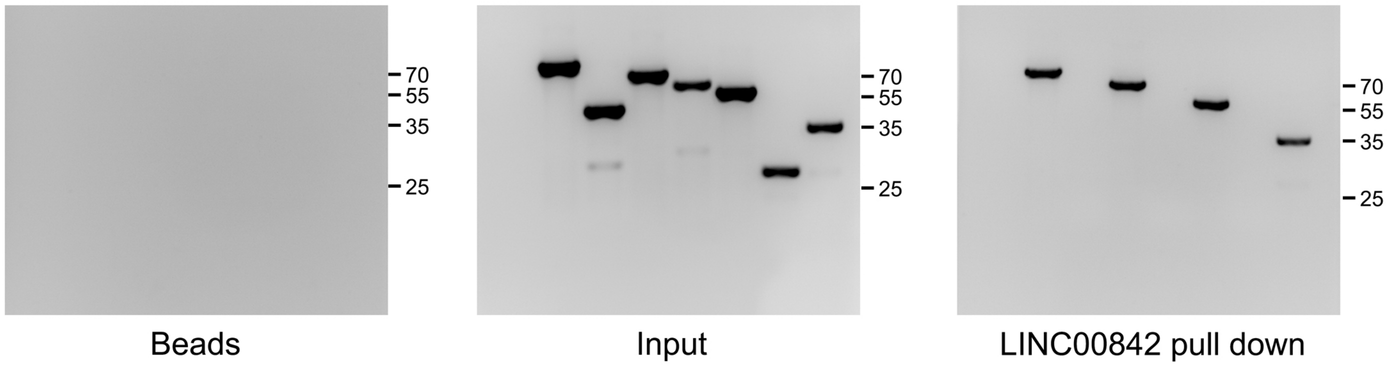

Figure 5a

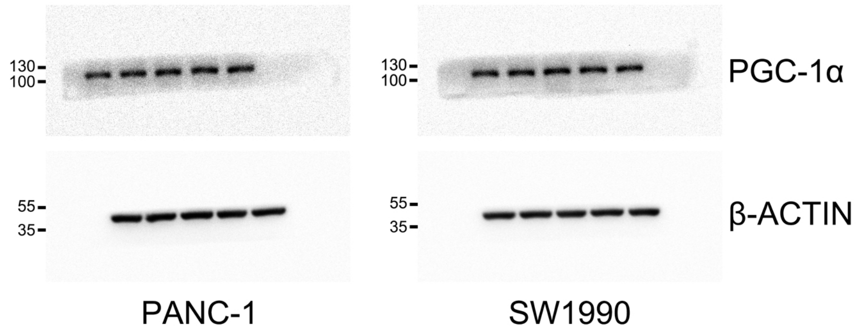

Figure 5b

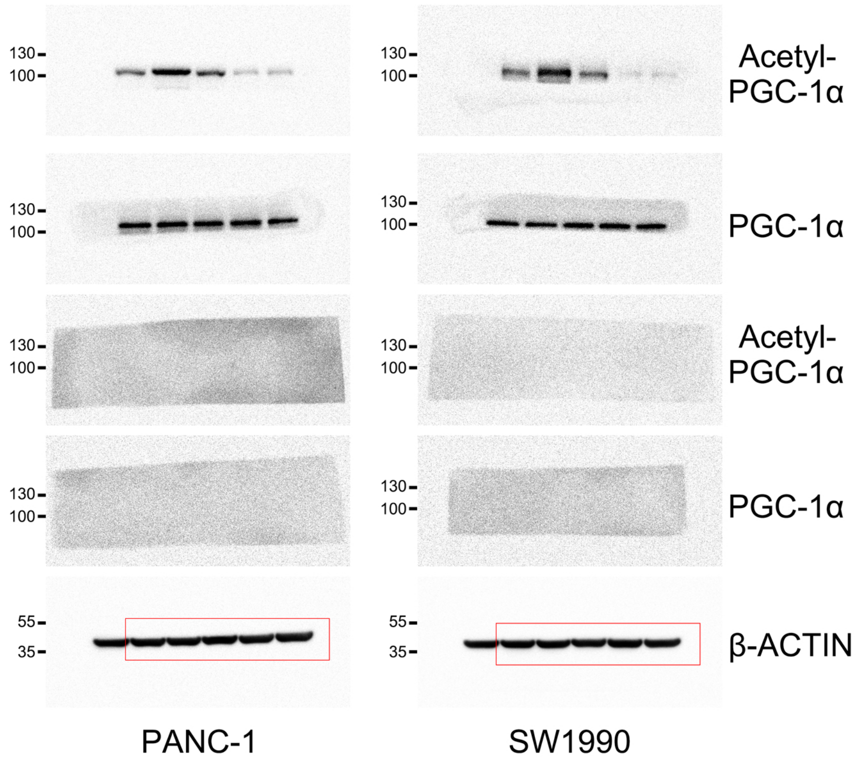

Figure 5c

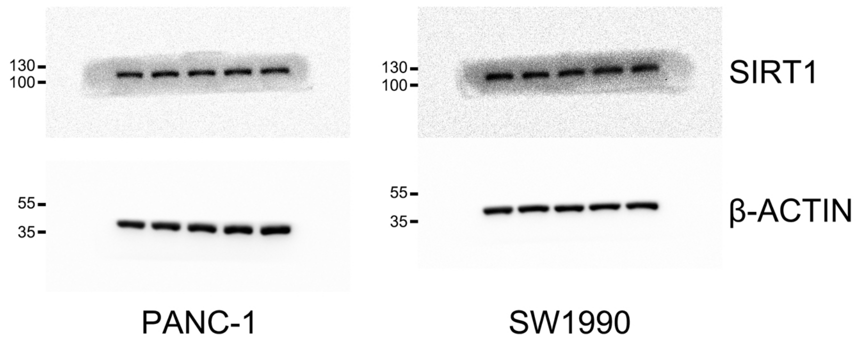

Figure 5h

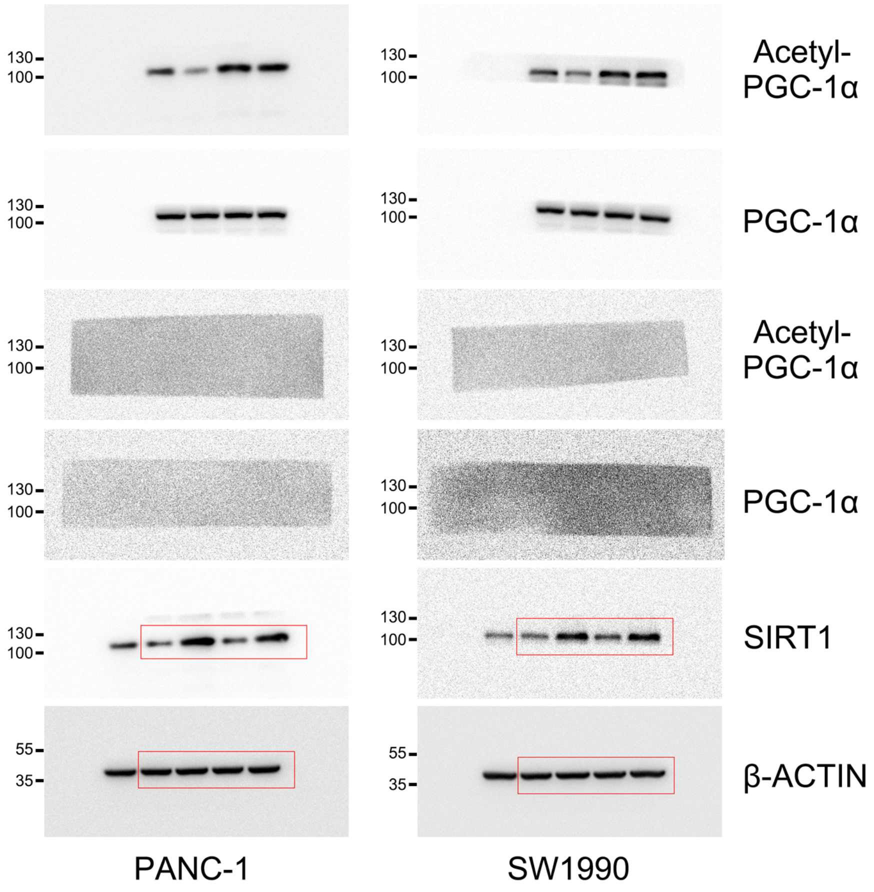

Figure 5d

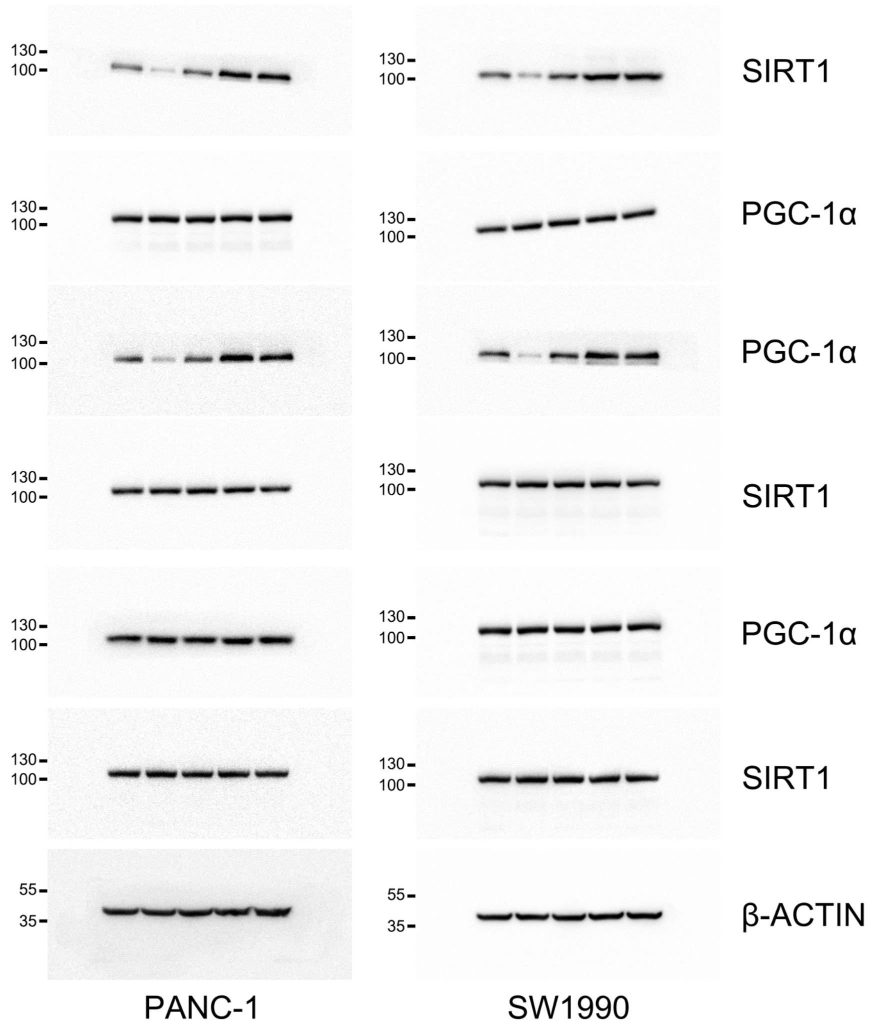

Figure 5g

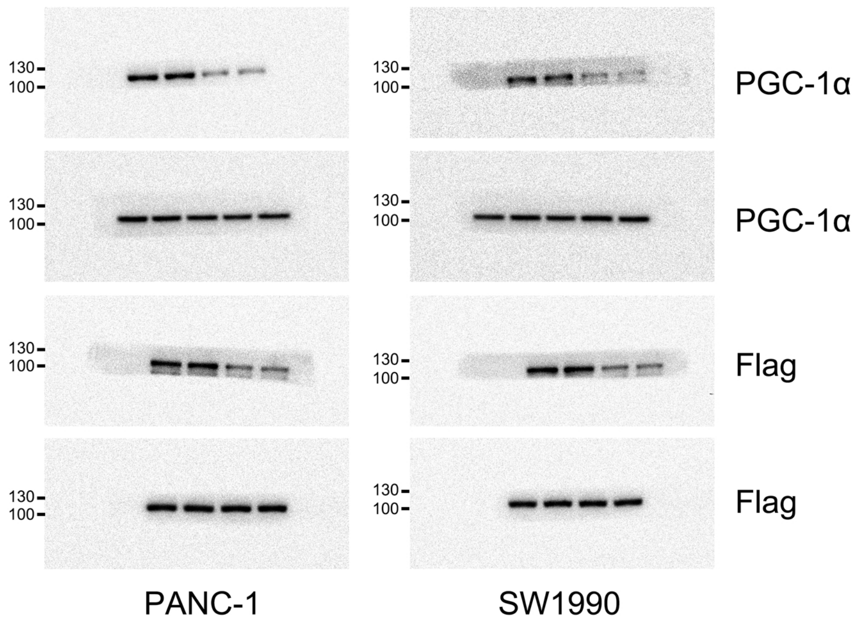

Figure 5i

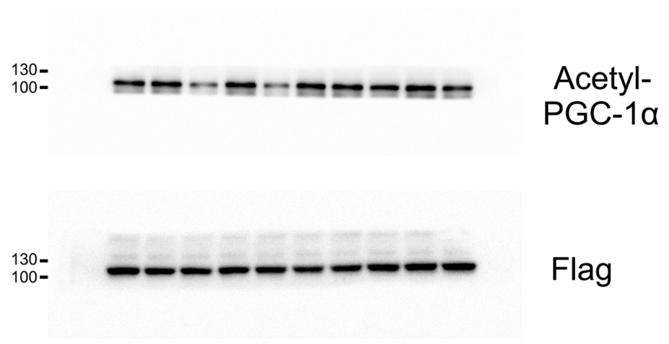

Figure 6e

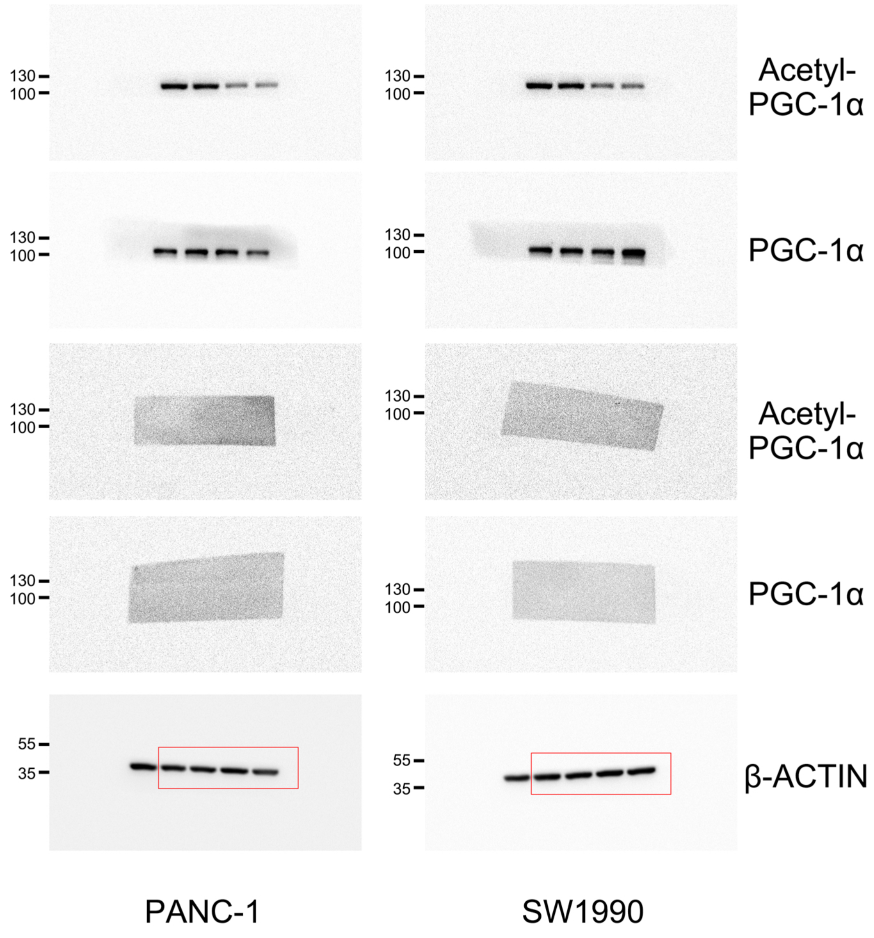

Figure 6f

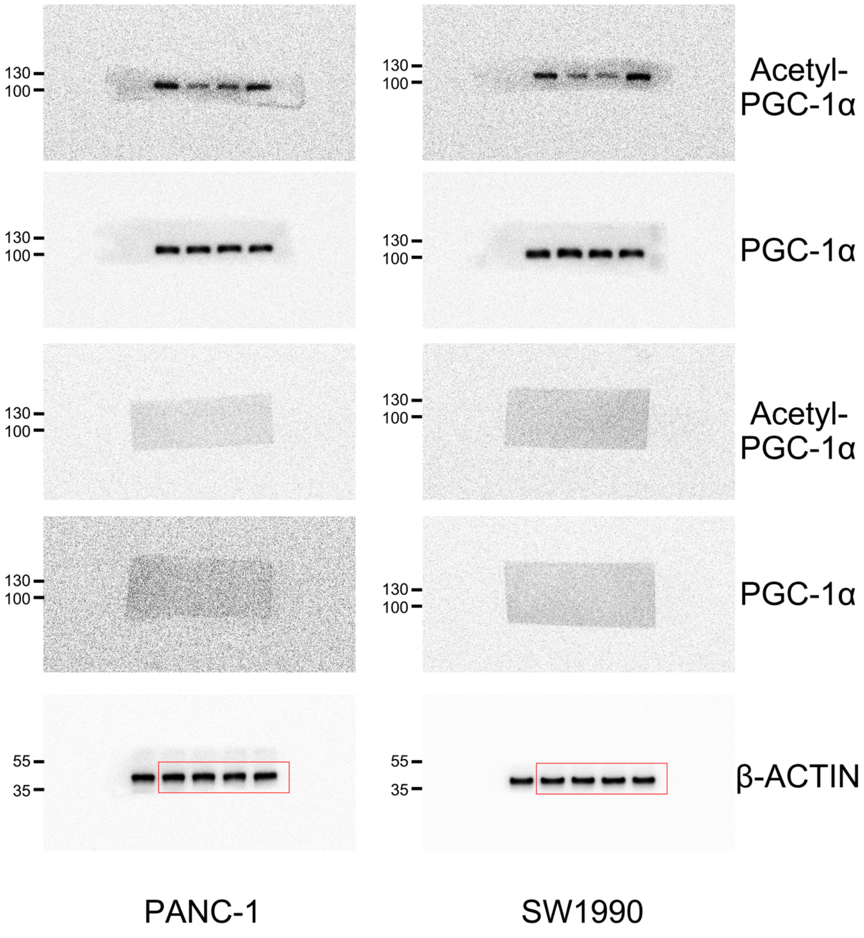

Figure 6g

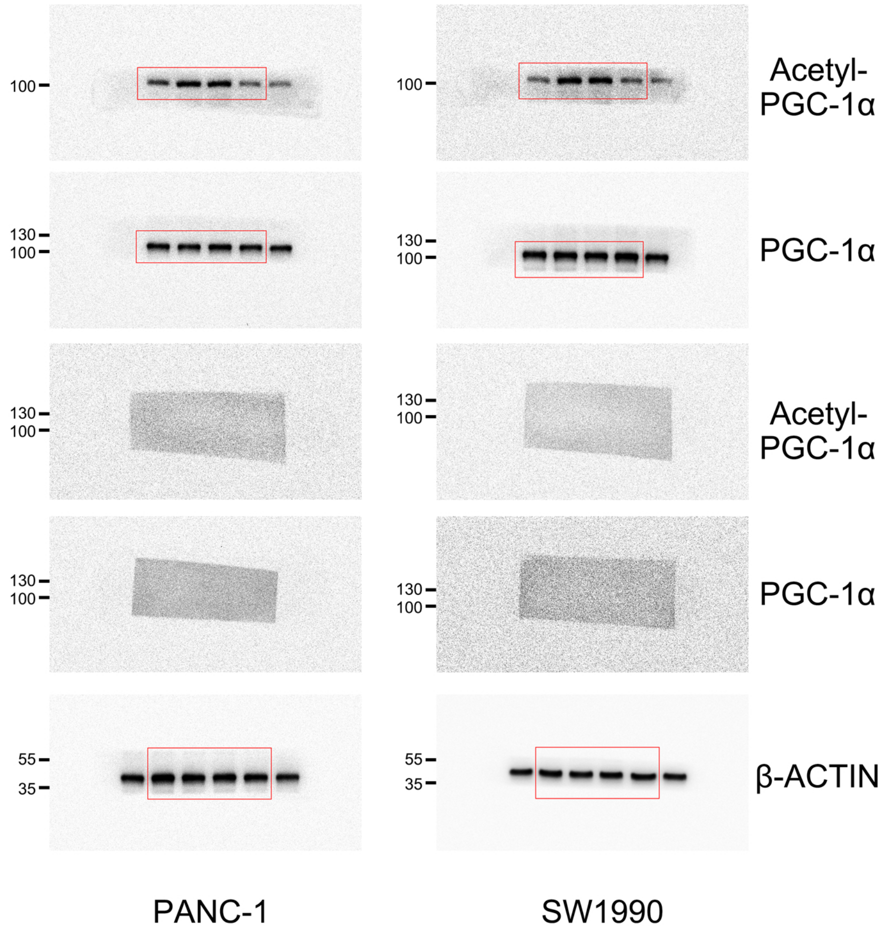

Figure 6h

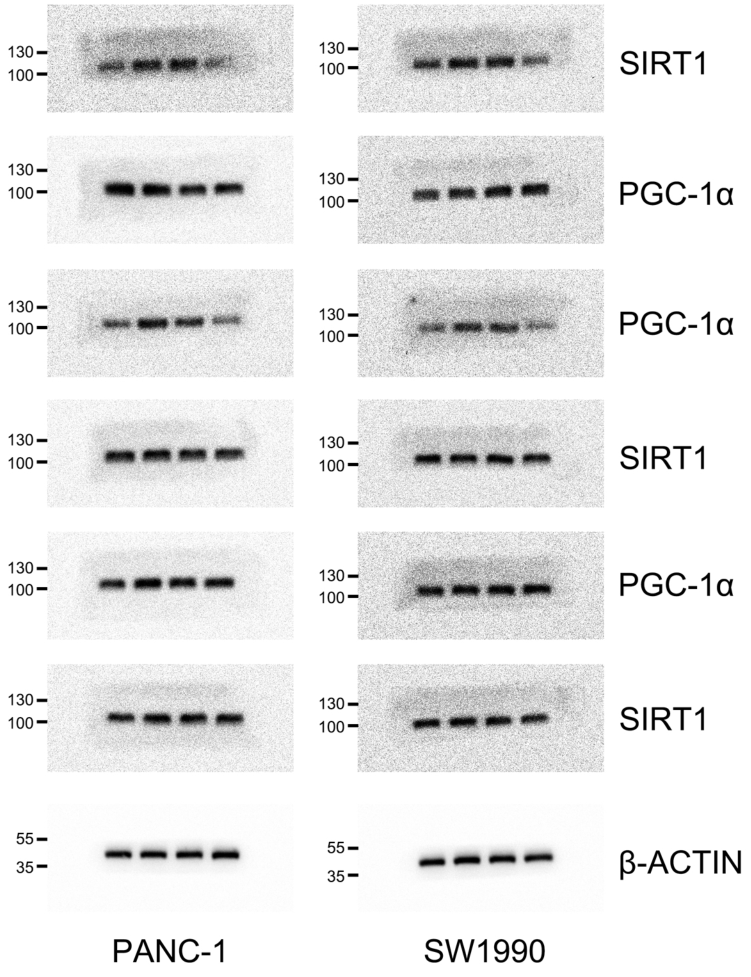

Figure 6i

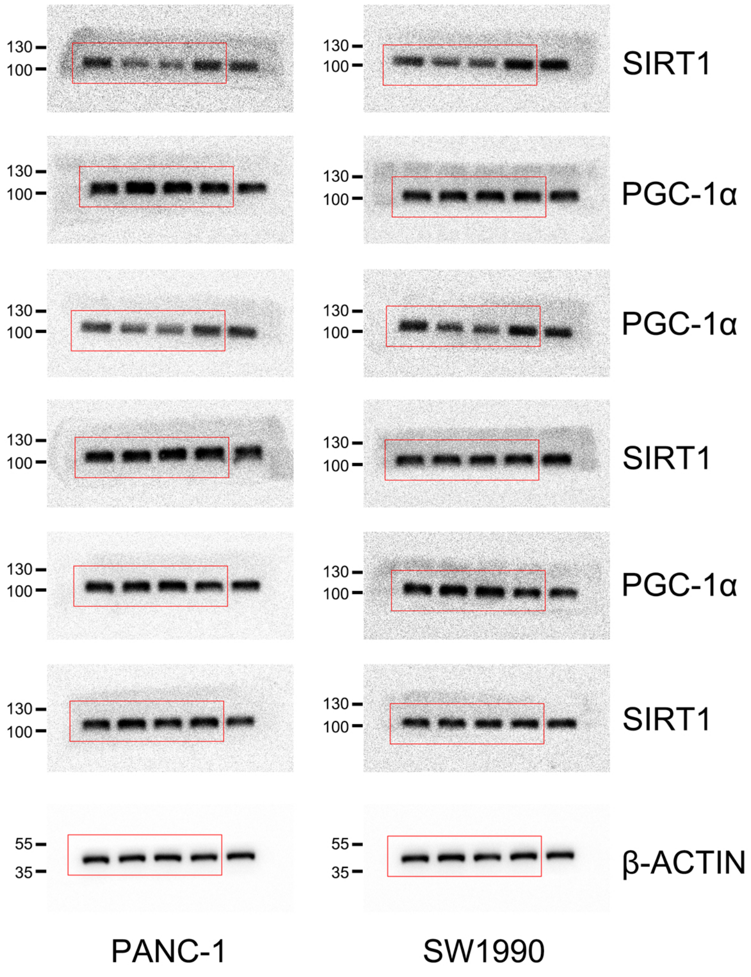

Figure 6m

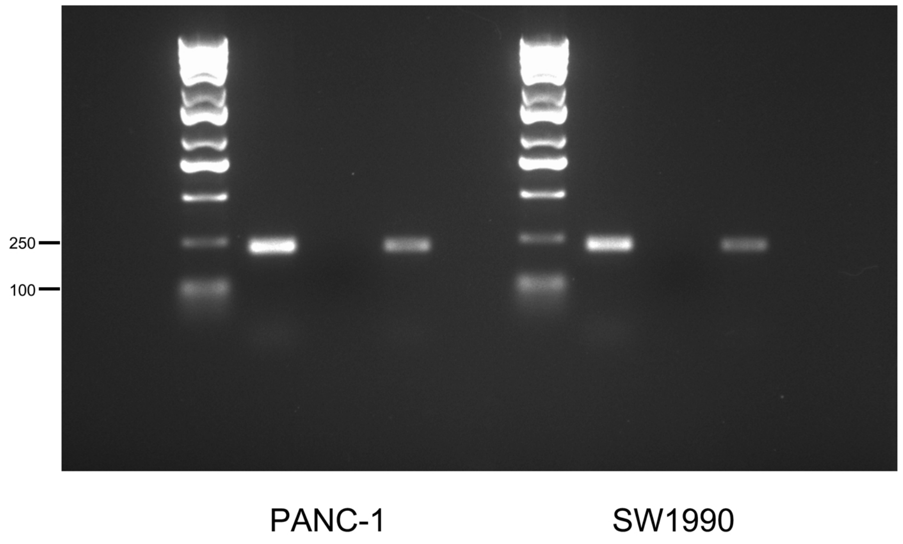

Figure 6n

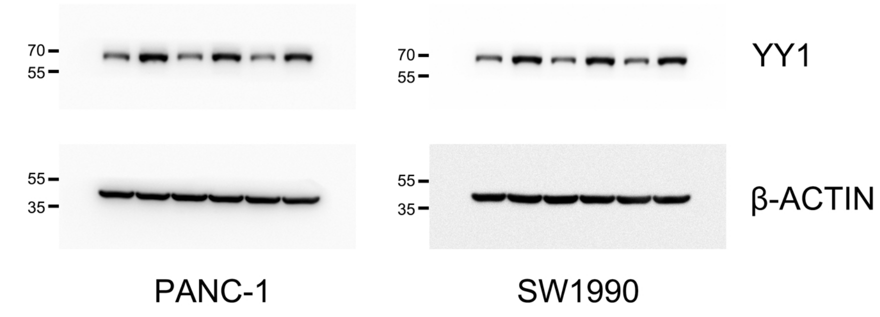

Figure 6p

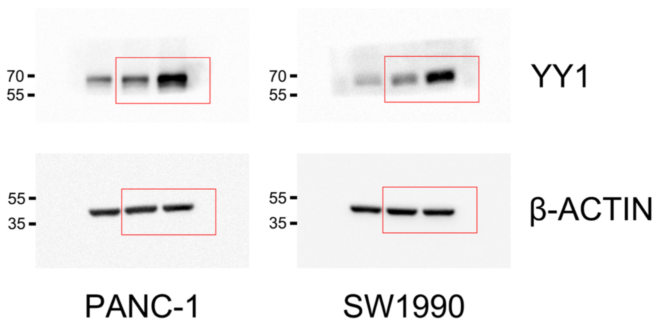

Figure 6q

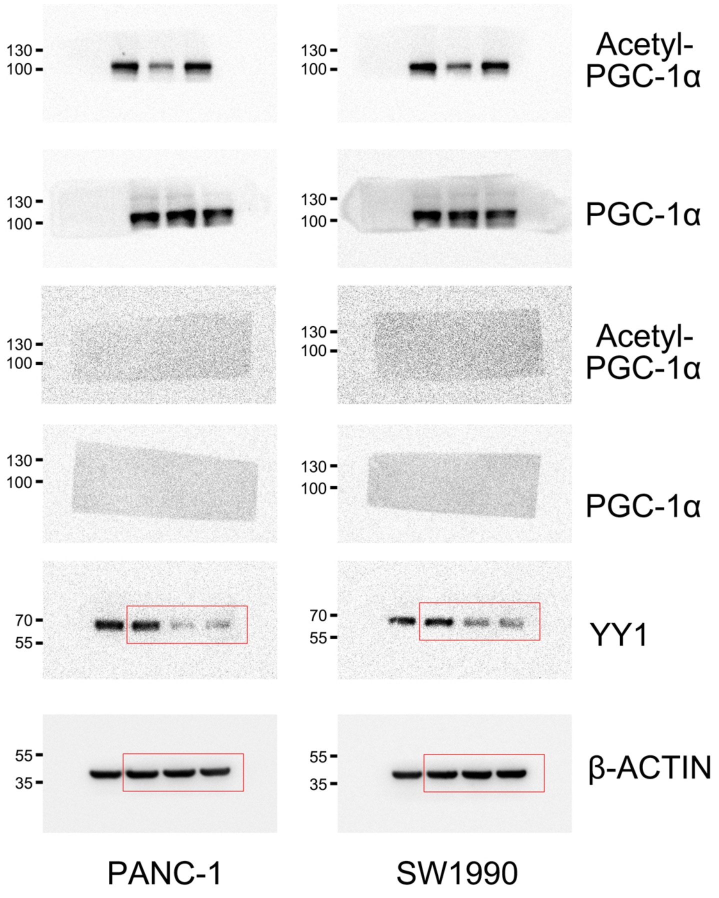

Figure 6r

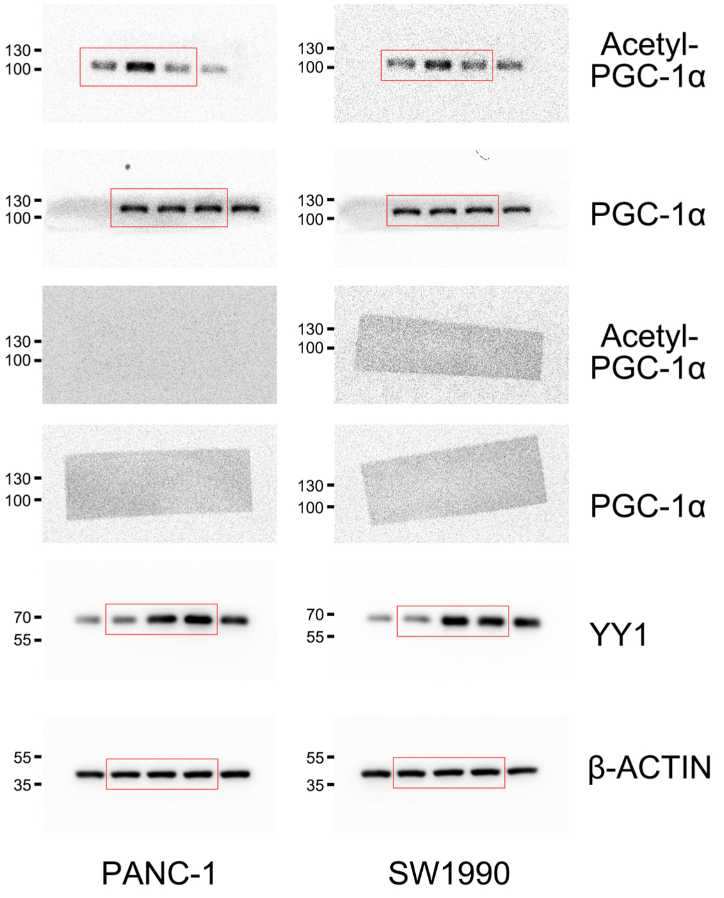

Figure 7a

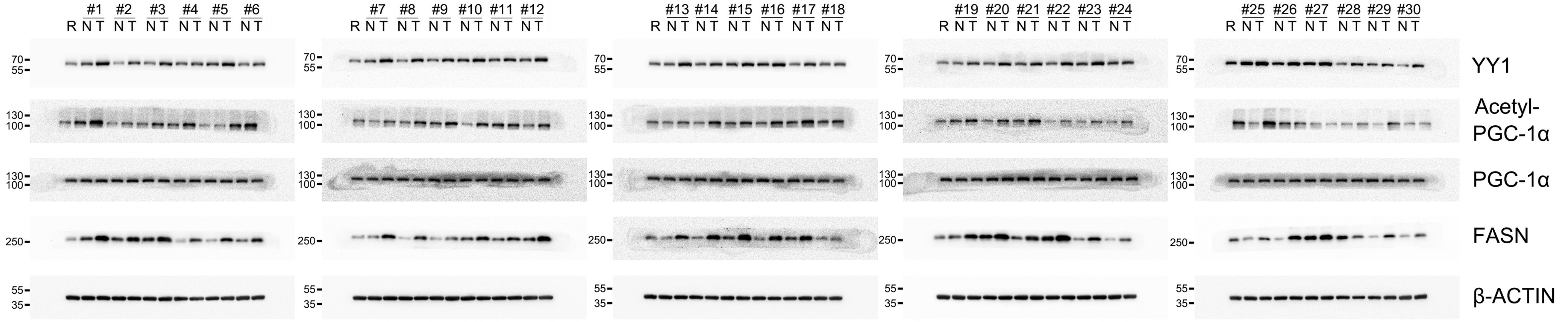
#1 #2 #3 #4 #5 #6
#7 #8 #9 #10 #11 #12
#13 #14 #15 #16 #17 #18
#19 #20 #21 #22 #23 #24
#25 #26 #27 #28 #29 #30
R N T N T N T N T N T N T
YY1
Acetyl-PGC-1α
PGC-1α
FASN
β-ACTIN
70
55
130
100
250
35

Supplementary Fig 1c

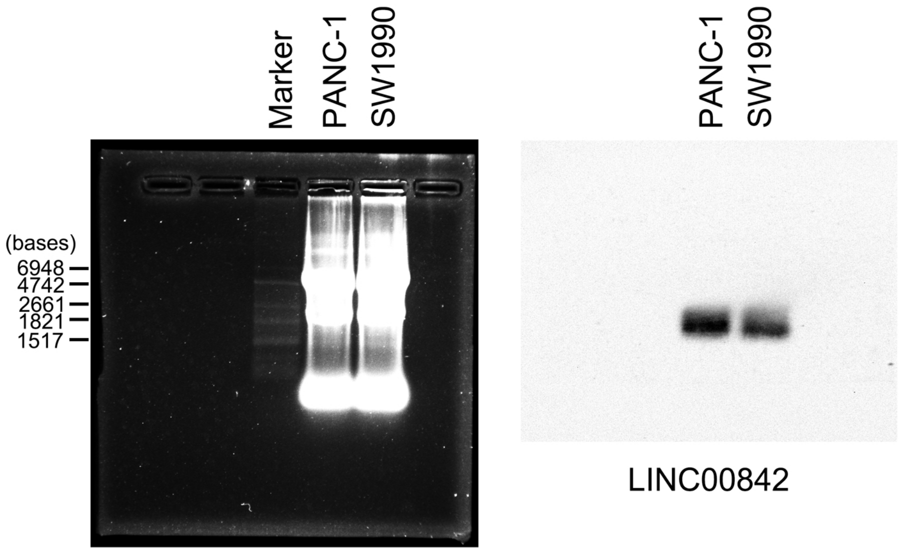

Supplementary Fig 1g

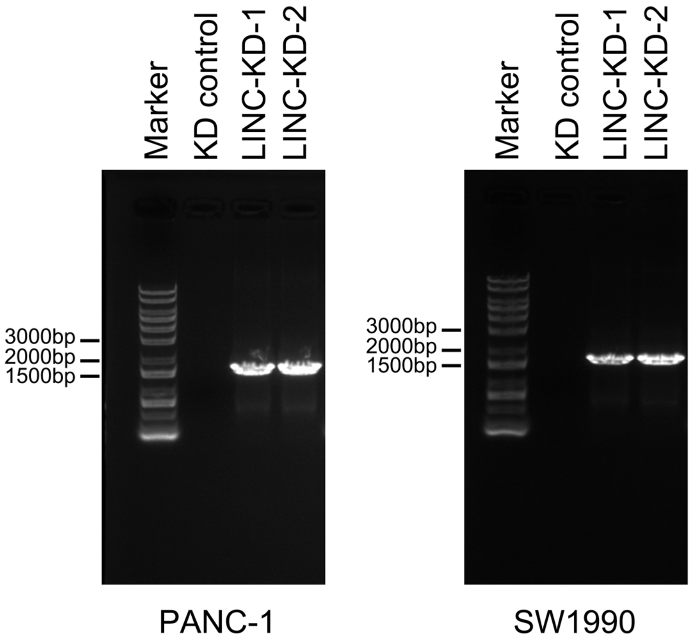

Supplementary Fig 5b

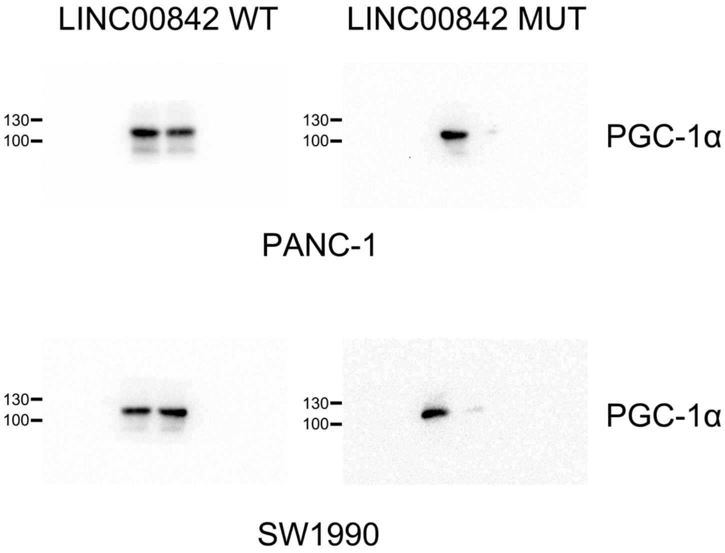

Supplementary Fig 6b

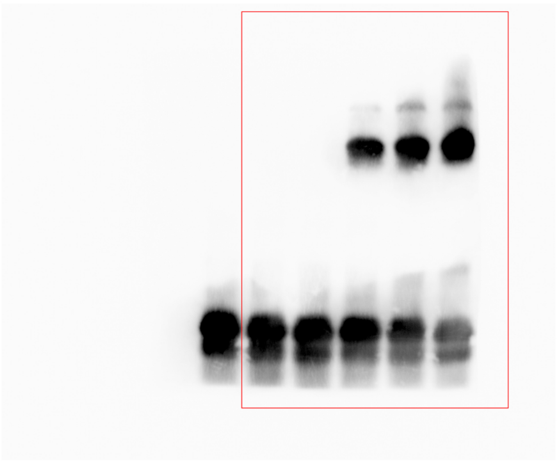

Supplementary Fig 6c

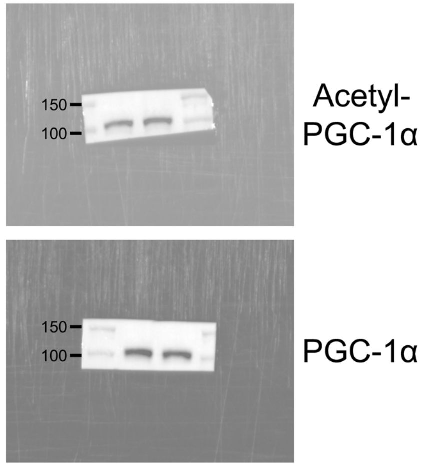

Supplementary Fig 6d

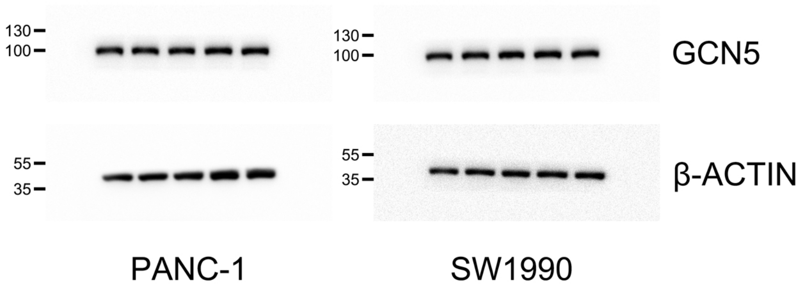

Supplementary Fig 6i

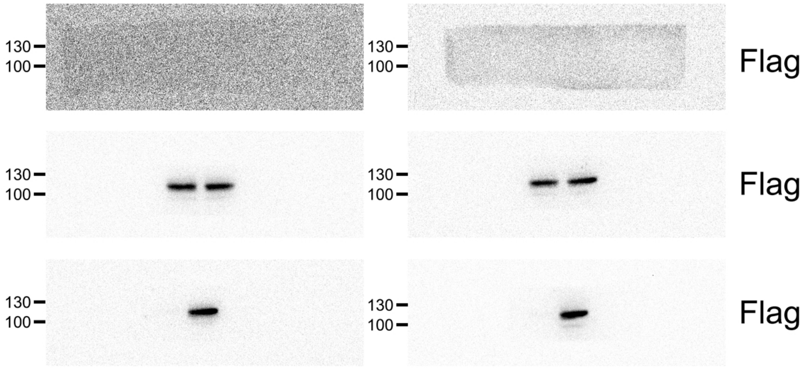

Supplementary Fig 6e

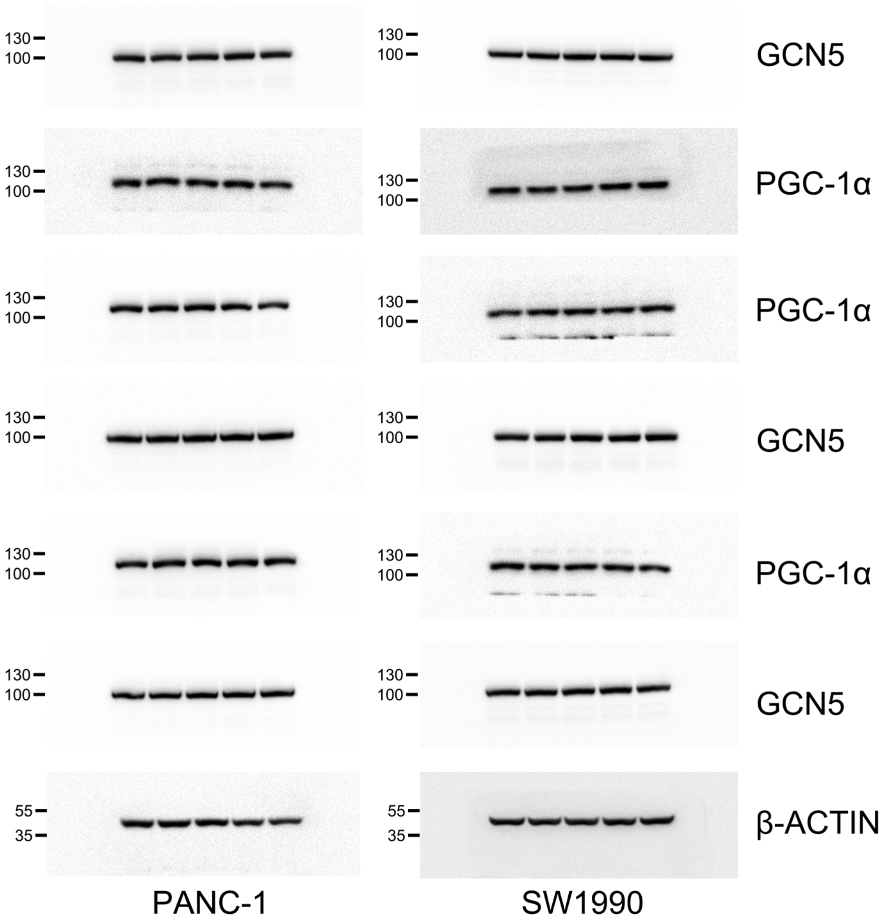

Supplementary Fig 6f

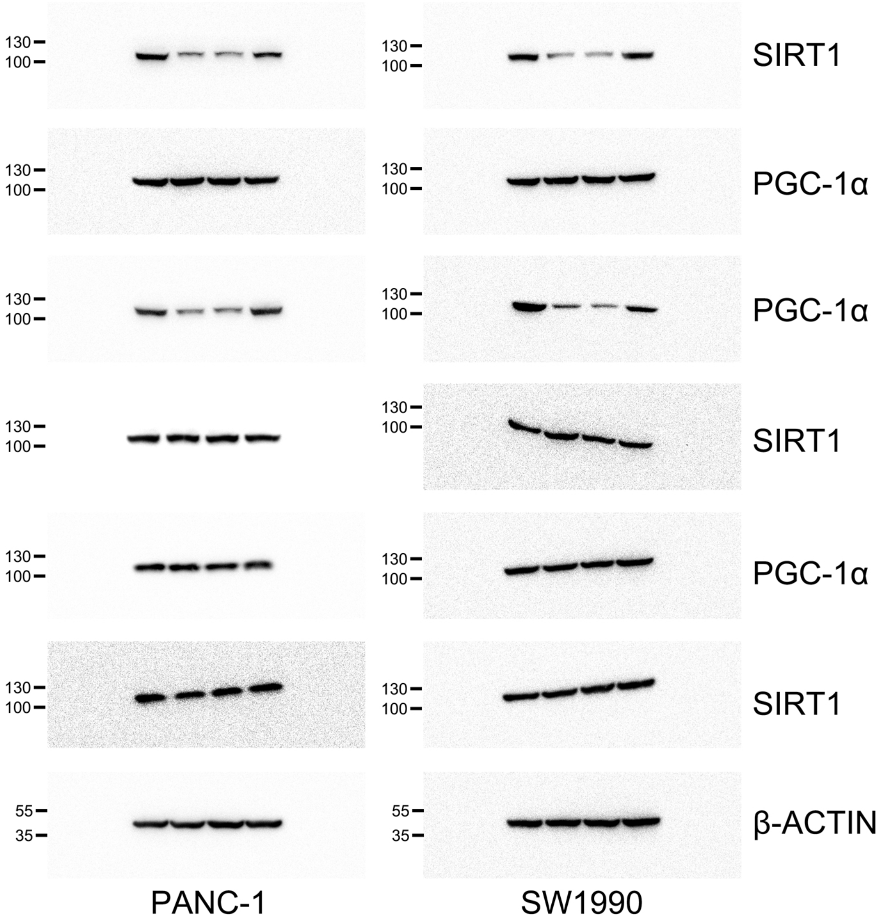

Supplementary Fig 6g

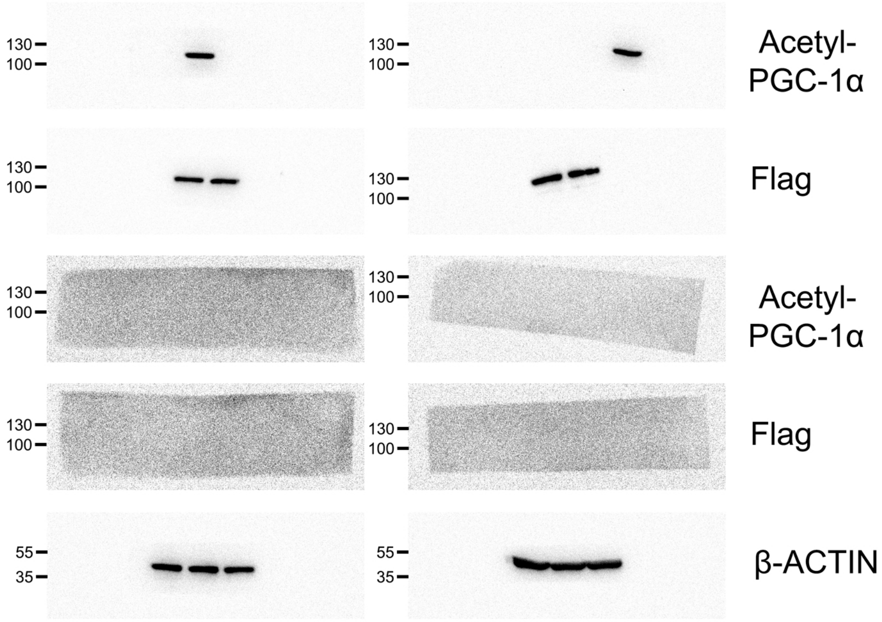

Supplementary Fig 14e

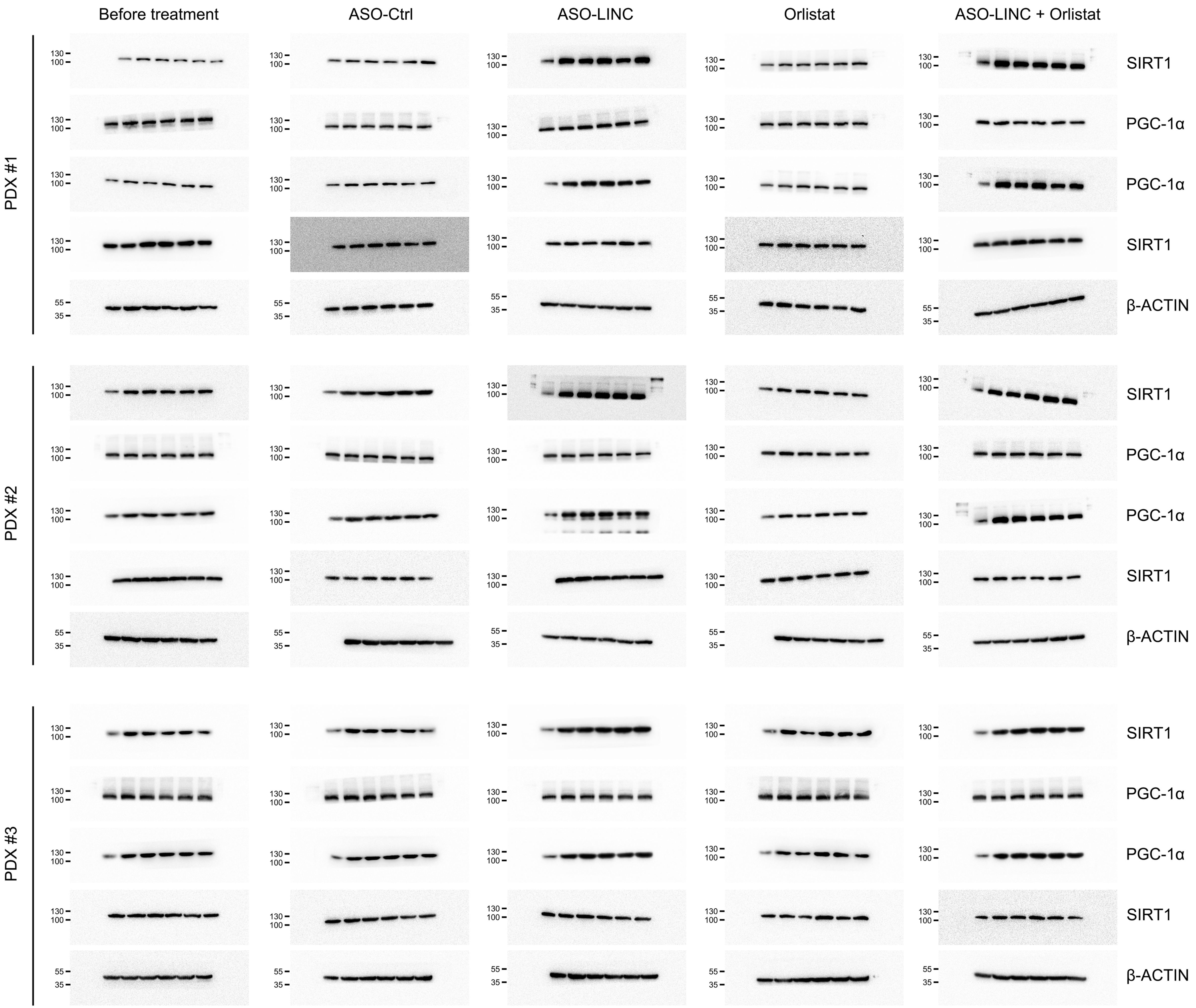

Supplementary Fig 14f

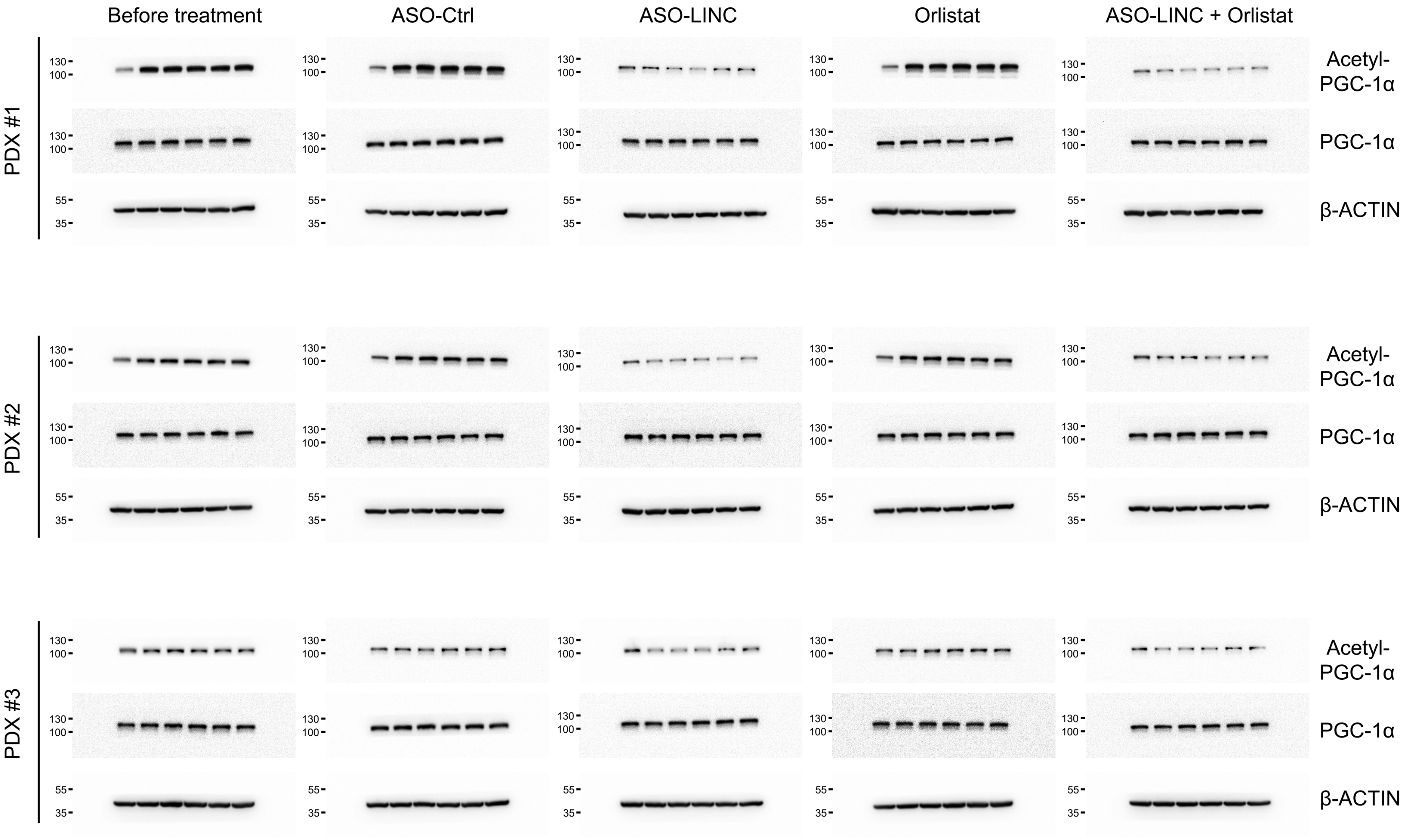

Supplementary Fig 15b

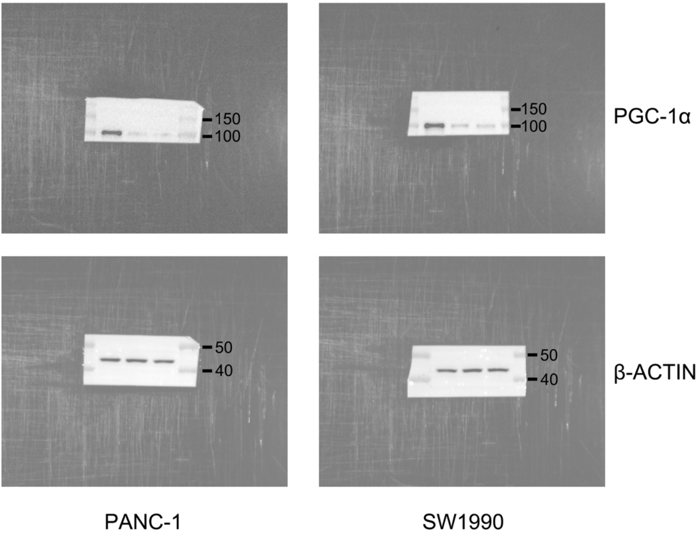

Supplementary Table 1. Sequences of sgRNAs, siRNAs or antisense oligonucleotides (ASOs) used in this study

**sgRNAs**	**Sequence (5'→3')**
sgRNA-Control	GCACTACCAGAGCTAACTCA
sgRNA-LINC00842	ATTCCGTGGAAGATGCCCAT
**ASO**	**Sequence (5'→3')**
ASO-LINC00842-1	CAAAGCCACTTCAACUGCAC
ASO-LINC00842-2	UUUGCTGACCTCCATGUCCU
**siRNAs**	**Sequence (5'→3')**
siControl	UUCUCCGAACGUGUCACGUTT
siPPARGC1A-1	GCUCGGAGCUUCUCAAAUATT
siPPARGC1A-2	GGACAGUGAUUUCAGUAAUTT
siBHLHE41-1	GCUGUAGUCUUGGAAUUAATT
siBHLHE41-2	CCGCACAGAUUAAUAGAAATT
siFOXC2-1	CUACCUGAGCGAGCAGAAUTT
siFOXC2-2	CUCCUACGACUGCACGAAATT
siSNAI2-1	CCGGAUACUCCUCAUCUUUTT
siSNAI2-2	GCUUCAAGGACACAUUAGATT
siPLAG1-1	GAGCCUUGGAAUGAGCUAUTT
siPLAG1-2	CCAGUGAACUGUUAUCAAATT
siTFAP2A-1	GGGUAUUAACAUCCCAGAUTT
siTFAP2A-2	GGAGACGUAAAGCUGCCAATT
siYY1-1	CCUGAAAUCUCACAUCUUATT
siYY1-2	GACGACUACAUUGAACAAATT

Supplementary Table 2. Proteins interacted with *LINC00842* identified by Mass Spectrometry (emPAI ≥ 0.10).

No.	Name and Official Symbol	Protein Mass	emPAI
1	Histone H2A type 1, HIST1H2AG	14083	1.38
2	Histone H1.4, HIST1H1E	21852	1.04
3	Peroxisome proliferator-activated receptor gamma coactivator 1-alpha, PPARGC1A	90971	0.96
4	Histone H2A.Z, H2AFZ	13545	0.96
5	Histone H2B type 2-F, HIST2H2BF	13912	0.93
6	rRNA 2~-O-methyltransferase fibrillarin, FBL	33763	0.76
7	Small nuclear ribonucleoprotein E, SNRPE	53222	0.74
8	Histone deacetylase 1, HDAC1	10055	0.42
9	Gamma-glutamylcyclotransferase, GGCT	20994	0.35
10	Complement component 1 Q subcomponent-binding protein, Mitochondrial, C1QBP	31343	0.35
11	Chromobox protein homolog 8, CBX8	43369	0.34
12	Protein S100-A6, S100A6	10173	0.34
13	Centromere protein W, CENPW	10055	0.34
14	Peroxiredoxin-2, PRDX2	21878	0.33
15	High mobility group nucleosome-binding domain-containing protein 3, HMGN3	10660	0.32
16	Catenin beta-1, CTNNB1	85442	0.30
17	Protein S100-A7, S100A7	11464	0.30
18	Small ubiquitin-related modifier 1, SUMO1	11550	0.30
19	E3 ubiquitin-protein ligase RING2, RNF2	37632	0.29
20	Small nuclear ribonucleoprotein-associated proteins B and B1, SNRPB	24594	0.29
21	Thioredoxin, TXN	11730	0.29
22	KH domain-containing, RNA-binding, signal transduction-associated protein 3, KHDRBS3	38776	0.28
23	Dynein light chain Tctex-type 1, DYNLT1	12444	0.28
24	Putative methyltransferase C9orf114, C9orf114	41982	0.26
25	Caspase-14, CASP14	27662	0.26
26	14-3-3 protein zeta/delta, YWHAZ	27728	0.25
27	14-3-3 protein sigma, SFN	27757	0.25

28	Exosome complex component MTR3, EXOSC6	28218	0.25
29	U2 small nuclear ribonucleoprotein A’, SNRPA1	28398	0.25
30	Suprabasin, SBSN	60505	0.24
31	Transcription initiation factor TFIID subunit 9, TAF9	28956	0.24
32	Extracellular glycoprotein lacritin, LACRT	14237	0.24
33	Galectin-7, LGALS7	15066	0.23
34	Serpin B12, SERPINB12	46247	0.23
35	Fatty acid-binding protein, epidermal, FABP5	15155	0.22
36	Mediator of RNA polymerase II transcription subunit 21, MED21	15555	0.22
37	Mediator of RNA polymerase II transcription subunit 10, MED10	15678	0.22
38	Putative uncharacterized protein encoded by LINC00479, LINC00479	15199	0.22
39	Septin-7, SEPT7	50648	0.21
40	Krueppel-like factor 5, KLF5	50759	0.21
41	Calmodulin-like protein 5, CALML5	15883	0.21
42	Nuclear pore glycoprotein p62, NUP62	53222	0.20
43	REST corepressor 1, RCOR1	52996	0.20
44	DNA topoisomerase I, mitochondrial, TOP1MT	69828	0.20
45	rRNA/tRNA 2’-O-methyltransferase fibrillarin-like protein 1, FBLL1	34782	0.20
46	Microsomal glutathione S-transferase 3, MGST3	16506	0.20
47	AP-2 complex subunit sigma, AP2S1	17007	0.20
48	Calmodulin, CALM1	16827	0.20
49	Mitotic interactor and substrate of PLK1, MISP	75311	0.19
50	Serine/threonine-protein phosphatase PP1-beta catalytic subunit, PPP1CB	37163	0.19
51	Serine/threonine-protein phosphatase PP1-gamma catalytic subunit, PPP1CC	36960	0.19
52	Septin-8, SEPT8	55721	0.19
53	Ubiquitin-associated protein 2-like, UBAP2L	114465	0.18
54	Polypyrimidine tract-binding protein 2, PTBP2	57455	0.18
55	Erlin-1, ERLIN1	38901	0.18
56	KH domain-containing, RNA-binding, signal transduction-associated protein 2, KHDRBS2	38904	0.18
57	Protein MAL2, MAL2	19113	0.18

58	SHC-transforming protein 1, SHC1	62782	0.17
59	T-complex protein 1 subunit alpha, TCP1	60306	0.17
60	Signal peptidase complex subunit 3, SPCS3	20301	0.17
61	ADP-ribosylation factor 6, ARF6	20069	0.17
62	Centrin-2, CETN2	19726	0.17
63	Putative cleavage and polyadenylation specificity factor subunit 4-like protein, CPSF4L	20713	0.17
64	5’-3’ exoribonuclease 2, XRN2	108513	0.16
65	Double-stranded RNA-binding protein Staufen homolog 1, STAU1	63143	0.16
66	Leucine-rich repeat-containing protein 47, LRRC47	63434	0.16
67	Translocon-associated protein subunit gamma, SSR3	21067	0.16
68	Core-binding factor subunit beta, CBFB	21495	0.16
69	DAZ-associated protein 1, DAZAP1	43356	0.16
70	Heat shock 70 kDa protein 6, HSPA6	70984	0.15
71	Cerebellin-4, CBLN4	21794	0.15
72	Claudin-4, CLDN4	22062	0.15
73	Aldehyde dehydrogenase, dimeric NADP-preferring, ALDH3A1	50363	0.14
74	Glial fibrillary acidic protein, GFAP	49850	0.14
75	Nucleolar and coiled-body phosphoprotein 1, NOLC1	73560	0.14
76	KHDC3-like protein, KHDC3L	24291	0.14
77	Desmin, DES	53503	0.13
78	Peripherin, PRPH	53618	0.13
79	Signal peptidase complex subunit 2, SPCS2	24987	0.13
80	Cardiotrophin-like cytokine factor 1, CLCF1	25160	0.13
81	Ribonuclease P protein subunit p29, POP4	25409	0.13
82	Homeobox protein Hox-C5, HOXC5	24960	0.13
83	Tetratricopeptide repeat protein 9B, TTC9B	25916	0.13
84	Aspartyl/asparaginyl beta-hydroxylase, ASPH	85809	0.12
85	DNA-binding protein SATB2, SATB2	82504	0.12
86	Protein HIRA, HIRA	111764	0.12
87	Neutral amino acid transporter B(0), SLC1A5	56562	0.12

88	T-complex protein 1 subunit zeta-2, CCT6B	57785	0.12
89	Runt-related transcription factor 2, RUNX2	56613	0.12
90	Leucine-rich repeat-containing protein 57, LRRC57	26737	0.12
91	Synaptophysin-like protein 1, SYPL1	28547	0.12
92	Complement factor D, CFD	27016	0.12
93	Paired mesoderm homeobox protein 1, PRRX1	27280	0.12
94	TATA-binding protein-associated factor 2N, TAF15	61793	0.11
95	Brain-specific angiogenesis inhibitor 1-associated protein 2, BAIAP2	60830	0.11
96	Polycomb group RING finger protein 5, PCGF5	29694	0.11
97	NAD(P)H dehydrogenase [quinone] 1, NQO1	30848	0.11
98	U1 small nuclear ribonucleoprotein A, SNRPA	31259	0.11
99	Interleukin-18 receptor 1, IL18R1	62264	0.11
100	Uncharacterized protein C2orf72, C2orf72	30462	0.11
101	Probable U3 small nucleolar RNA-associated protein 11, UTP11L	30428	0.11
102	28S ribosomal protein S15, mitochondrial, MRPS15	29823	0.11
103	F-box only protein 44, FBXO44	29728	0.11
104	Pre-mRNA 3’-end-processing factor FIP1, FIP1L1	66487	0.10
105	Aryl hydrocarbon receptor nuclear translocator-like protein 1, ARNTL	68718	0.10
106	Menin, MEN1	67981	0.10
107	Very-long-chain 3-oxoacyl-CoA reductase, HSD17B12	34302	0.10
108	Malectin, MLEC	32214	0.10
109	Arginase-1, ARG1	34713	0.10
110	Metastasis-associated protein MTA3, MTA3	67461	0.10
111	U3 small nucleolar ribonucleoprotein protein IMP4, IMP4	33736	0.10
112	Transcriptional activator protein Pur-beta, PURB	33220	0.10
113	Tumor necrosis factor ligand superfamily member 10, TNFSF10	32488	0.10
114	Polyadenylate-binding protein 2, PABPN1	32729	0.10
115	26S proteasome non-ATPase regulatory subunit 14, PSMD14	34555	0.10
116	NK1 transcription factor-related protein 2, NKX1-2	32362	0.10

Supplementary Table 3. Baseline demographic and clinical characteristics of individuals with pancreatic ductal adenocarcinoma (PDAC) in this study

	Cohort 1 (Guangzhou)		Cohort 2 (Beijing)		Pooled samples	
	Alive (n = 36)	Deceased (n = 122)	Alive (n = 13)	Deceased (n = 56)	Alive (n = 49)	Deceased (n = 178)
**Age, mean (SEM[a])**	59.8 (2.0)	61.1 (1.0)	60.1(1.9)	63.1 (1.5)	59.9 (1.5)	61.7 (0.8)
**Sex, n (%)**						
Male	23 (63.9)	68 (55.7)	6 (46.2)	35 (62.5)	29 (59.2)	103 (57.9)
Female	13 (36.1)	54 (44.3)	7 (53.8)	21 (37.5)	20 (40.8)	75 (42.1)
**Differentiation, n (%)**						
Well	7 (19.4)	13 (10.7)	3 (23.1)	6 (10.7)	10 (20.4)	19 (10.7)
Moderate	22 (61.2)	81 (66.4)	8 (61.5)	31 (55.4)	30 (61.2)	112 (62.9)
Poor	7 (19.4)	28 (22.9)	2 (15.4)	19 (33.9)	9 (18.4)	47 (26.4)
**Lymph node metastasis, n (%)**						
Positive	14 (38.9)	66 (54.1)	7 (53.8)	27 (48.2)	21 (42.9)	93 (52.2)
Negative	22 (61.1)	56 (45.9)	6 (46.2)	29 (51.8)	28 (57.1)	85 (47.8)
**Vascular invasion, n (%)**						
Yes	10 (27.8)	39 (32.0)	5 (38.5)	27 (48.2)	15 (30.6)	66 (37.1)
No	26 (72.2)	83 (68.0)	8 (61.5)	29 (51.8)	34 (69.4)	112 (62.9)
**Neural invasion, n (%)**						
Yes	23 (63.9)	74 (60.7)	3 (23.1)	10 (17.9)	26 (53.1)	84 (47.2)
No	13 (36.1)	48 (39.3)	10 (76.9)	46 (82.1)	23 (46.9)	94 (52.8)
**TNM stage[b], n (%)**						
I	1 (2.8)	6 (4.9)	3 (23.1)	9 (16.0)	4 (8.2)	15 (8.4)
II	29 (80.6)	100 (82.0)	8 (61.5)	27 (48.2)	37 (75.5)	127 (71.3)
III	0 (0.0)	4 (3.3)	2 (15.4)	10 (17.9)	2 (4.1)	14 (7.9)
IV	6 (16.7)	12 (9.8)	0 (0.0)	10 (17.9)	6 (12.2)	22 (12.4)
**Smoking status, n (%)**						
Ever	8 (22.2)	44 (36.1)	3 (23.1)	18 (32.1)	11 (22.4)	62 (34.8)
Never	28 (77.8)	78 (63.9)	10 (76.9)	38 (67.9)	38 (77.6)	116 (65.2)
**Drinking status, n (%)**						
Ever	5 (13.9)	21 (17.2)	4 (30.8)	16 (28.6)	9 (18.4)	37 (20.8)
Never	31 (86.1)	101 (82.8)	9 (69.2)	40 (71.4)	40 (81.6)	141 (79.2)
**Treatment, n (%)**						

Surgery only	13 (36.1)	69 (56.6)	6 (46.2)	26 (46.4)	19 (38.8)	95 (53.4)
Surgery + chemotherapy	23 (63.9)	53 (43.4)	7 (53.8)	30 (53.6)	30 (61.2)	83 (46.6)

[a]SEM, standard error of mean.

[b]Tumor TNM staging were reviewed by at least 3 pathologists and defined according to the American Joint Committee on Cancer (AJCC) 8th edition.

Supplementary Table 4. Primers and probes used for *in vitro* transcription, ChIRP, qRT-PCR, ChIP-qPCR or Northern blot analysis in this study.

***In vitro* transcribed LINC00842 and its truncated fragments**	**Sequence (5'→3')**
LINC00842-F[a]	TAATACGACTCACTATAGGGAGAGCGGGGGCGGGCGCG
LINC00842-R	TCTATGAGCTTTTCCTTTATTAGGG
1-2354-R	TTCCTAACCAACGGTACCTCATCA
1-1796-R	TTCCTAACCAACGGTACCTCATCA
1-1242-R	TGTCTTGATCTCTAGAGAGAGCATT
1-690-R	TCACAACCCCTGCAGCAGGCGGGA
691-2872-F[a]	TAATACGACTCACTATAGGGAGATGTATCAGAACCTTGAAGAG
691-2872-R	TCTATGAGCTTTTCCTTTATTAGGG
Antisense LINC00842-F[a]	TAATACGACTCACTATAGGGAGATCTATGAGCTTTTCC
Antisense LINC00842-R	GCGGGGGCGGGCGCGC

**ChIRP probes (3'-biotin)**	**Sequence (5'→3')**
LINC00842-1	TCAACACAACGCGTCACTCC
LINC00842-2	AGCGCTCCAGAAAACTGGTG
LINC00842-3	GCTCAGCTGTTAAGAACACA
LINC00842-4	ACCTGATTGGAGAAACAGAC
LINC00842-5	TGCAGTTGAAGTGGCTTTGA
LINC00842-6	CAAGGTGTGTCTTTTTCGTC
LINC00842-7	GTTTTTTTCTCACGGCTTAA
LINC00842-8	TTCCAGAAACTGGACTCCTG
LINC00842-9	CTTGCTACTTCGATTCTTGG
LINC00842-10	GAGACTTGTGCCTTAACTGG
LINC00842-11	TTCTGATACACCTCACAACC
LINC00842-12	ATCTGATTCTTACAGCGCTG
LINC00842-13	GCAGGGATACAGTGATGACT
LINC00842-14	TGTTGCTGTGGTTCACTGAG
LINC00842-15	TCAAGCTTCTCGGCTGGAAG

LINC00842-16	CTTCTGTAGAGAATCGAGGC
LINC00842-17	GATGATACCAGGTAAGTGGC
LINC00842-18	TAGTTCTCCAGAGCTGAGAC
LINC00842-19	AAGGAGGTCTTGGTGGACAG
LINC00842-20	AACAAGTTAGGGTCCTGAGG
LINC00842-21	TGAGAGGACATGGAGGTCAG
LINC00842-22	TAGAGAGAGCATTGGCCAAG
LINC00842-23	GATGGTAGGGCTCTCTAGTC
LINC00842-24	TCAGTCAGGCACTCAGTAAC

**Gene Symbol**	**qRT-PCR sequence (5'→3')**	
	**Forward**	**Reverse**
ZNF667-AS1	GTTTGAGGTGACCGTTGCGTA	CTACACAAACGCGCGATCAA
FAM83A-AS1	AGCAGGGCTCTTCAGTTTGG	GCAGGGCCGTCTGTGTTTAC
ATP2B1-AS1	TTGTTTCTTCTACTATGCCAAGC	ACCGCAAGTTATCCCGTTT
STXBP5-AS1	TCTGAAGGAGAAAGTTATCTGAGGC	ATAGAGACGGAAAGTAGAATGGTGT
LINC00996	TCTCTGCCCCTTTGCACATG	GGAGCCTACACAGCACCCCT
LINC00842	TCAGGACTGGAGGGGCAAAC	AGCAGGCGGGAGACTTGTG
LINC00941	TTGATAGCCAAACAACAGT	AATATGGAGGCTGAGAAGT
RAMP2-AS1	CAAAACCAGGGAGGAGAAGC	CATGAAGTCGCACACTGAAGG
UBAC2-AS1	CTGGTCACTCAGATGCGGATAG	AGGGTAGGGCTTGGCACTTTC
ABHD11-AS1	CACTGGGGGACACCCGGACA	CCTGCTTCTTGGCAATGGCTTCA
MDH1	TTTGGATCACAACCGAGCTAAAG	ACATCTGGATACTGAGTCGAGG
PDHB	AGTGGTGGTGCTAGAGAATGA	TGCAGCTTCTAAGCAGTGGC
SUCLG1	TATGGCACCAAACTCGTTGGA	GAAGCCGTTGCTCCTGTCT
DLAT	CCGCCGCTATTACAGTCTTCC	CTCTGCAATTAGGTCACCTTCAT
IDH3A	CCCGCGTGGATCTCTAAGG	AATTTCTGGGCCAATACCATCTC
CS	TGCTTCCTCCACGAATTTGAAA	CCACCATACATCATGTCCACAG
SDHA	CAGCATGTGTTACCAAGCTGT	GGTGTCGTAGAAATGCCACCT
FH	GGAGGTGTGACAGAACGCAT	CATCTGCTGCCTTCATTATTGC

GOT1	ATTTCTTAGCGCGTTGGTACA	ACACAGCATTGTGATTCTCCC
MPC1	ATTTGCCTACAAGGTACAGCC	AGTCATCTCGTGTTTGATAAGCC
MPC2	TACCACCGGCTCCTCGATAAA	TATCAGCCAATCCAGCACACA
ACADM	GGAAGCAGATACCCCAGGAAT	AGCTCCGTCACCAATTAAAACAT
ACAT1	ATGCCAGTACACTGAATGATGG	GATGCAGCATATACAGGAGCAA
CPT1A	ATCAATCGGACTCTGGAAACGG	TCAGGGAGTAGCGCATGGT
SLC25A1	CTGGAGGCGCACAAATACCG	GAGCAGCTTCACCACTTCATCATAG
TECR	TCTACTTCCGAGTGCCCTTCA	GCTTGATGTAGTGGAATGAGTGA
FASN	AAGGACCTGTCTAGGTTTGATGC	TGGCTTCATAGGTGACTTCCA
SREBF1	CGGAACCATCTTGGCAACAGT	CGCTTCTCAATGGCGTTGT
ATP5F1	ATTAGCGCAGAGACCTTCACT	CGCCTCTTCTAGTTGGGCAAG
U6	CTCGCTTCGGCAGCACA	AACGCTTCACGAATTTGCGT
GAPDH	TTGGCCAGGGGTGCTAAG	AGCCAAAAGGGTCATCATCTC
KAT2A	GCAAGGCCAATGAAACCTGTA	TCCAAGTGGGATACGTGGTCA
SIRT1	TAGCCTTGTCAGATAAGGAAGGA	ACAGCTTCACAGTCAACTTTGT
PPARGC1A	TGAAGACGGATTGCCCTCATT	GCTGGTGCCAGTAAGAGCTT
BHLHE41	TAACCGAGCAACAGCATCAGA	CATGTTTGAAATCCCGAGTGG
FOXC2	CCTCCTGGTATCTCAACCACA	GAGGGTCGAGTTCTCAATCCC
SNAI2	CAAAAAGCCAAACTACAGCGAAC	GTGGTATGACAGGCATGGAGTAAC
PLAG1	AAACTTTTGAAAGCACGGGAGT	GGCGATCACAATGTTCGCAC
TFAP2A	TTAACATCCCAGATCAAACTGTAAT	ACCCGGAACTGAACAGAAGACT
YY1	CCTCTCAGATCCCAAACAACTG	GCCTTTATGAGGGCAAGCTATT
β-ACTIN	CAGGGCGTGATGGTGGGCATG	GTAGAAGGTGTGGTGCCAGATT

**ChIP-qPCR**	**sequence (5'→3')**	
	**Forward**	**Reverse**
LINC00842-promoter	CCACTCTTTCAGTAGCTCCTTCA	CCTAAACATGCCAAGTCCAACA

LINC00842 Northern blot probe sequence (5'→3')

DIG-TCCATCGGTTTCCATATTCCCTTGGCAAAGGTTCGTACATGGAGCATTACCTCCCCACACTCTGG
GGACACACAGGAACACTGAACATACCTGGACATAAACAGAGCAGGTCCTGCAGAGATGAGAAGGAT

GTTTGGGGTATGAGGTGAGGCGCCATCAGGTCGCACCTGTGTGAAGCCAGTGAGCTCTCTCAGAGC
TGGTGCCTCAGTGGGTGCTGGACCAAGAGGCCAGTGAGGACAAGAGGATCATAAGCAGAGCAGAA
ACAGCATCCAATTCAGGGTCAGAGGAGGTGGTGTGGGCTGAACTGTGTCCCCCCAAACTCATATATT
GAATTCCTAACCAACGGTACCTCATCATGTGGCCTTCTTTGGAAGCAGTGTCATTGCAGGTATAATTAG
TTAAGATGAGGTCATACTGGAATATAGTGGGTCCCTAATCCAATCCCTTGGCAAAGGTTCATACATTGA
GCATTGTCCGTATAAGACTGGTGTCCTTATAAGAAGGGGAAATTTGGACACAGAAACACACACAGGG
AGGATGTGTTGTGACCATGAAGGCAGAGTTCAGGGTAGTCCTTCTACAAGCCAAGGCATGCTGAAG
ATGGAAGTAATCCACTAGAAGCTGAGGGACAGGCACCCAACAGGCTCCTGCACAGCCTTCCGAAGG
AACCAACCCTGCTGACACCTTGACCTTGAACTTGCAGCCTCCAGAACTGTGAGACCATAAGTTTCTGT
CGTTTTAGCCGCTCTGCCTGTAACGCTTTGTTGTGGCAACCCCAGGAAACTAACAGAGGAGGTGAGA
TTGGGGGCAGGGAGGCCCATCTGTGGGGGGCATCATGTCAGTCAGGCACTCAGTAACTGGGGGAA
AATCCCAAACTCAAGTGGGTGATGGCAAAATGGGGAAATTATCGGCTCATGGAATGGGGCTCTCCAA
AGGAAGTTCTTTGACTTCAGGCTTGATTCAAGGGTTTGAACGAGGATGGTAGGGCTCTCTAGTCCTT
GCCCCCTTGTCTCCACATGTTTTGAACTGCACGTTTGTGTGCCTCCAGAATTCCTATGTTGAAACCCTA
ACCCACAATGGGATGGTATGTGGAGATGGAGCCTTTGAGAAGTGATTAGATTTAGTTGAAGTTATGG
GGATTCTTCATGACGGGATTAGGCCCTTACCAGAAGAGACCAAAGAGCATGCTCTCTCTCTCTCTC
TCTCTTTCTCTGTCTTTCTGTCTCTCTCTCTCTCTCTCTGCCATGTGAAGATACATCAGGGAGATGTC
CATCTGCAACCCAGGAAGAGAGCCCTCACGAGGAAACAAGCAGGCCAGTACCCCAGTCTGTAGGAC
TTCCCAGCCTCCAGAACAGTGAGAAATAAATTTCTGTGGTTTATAAGCCACCCAGCTTAAGGCATTTT
GTCACAGCAGCCAGAACAGACCAAGAGAGGTGCCTGTGTCTTGATCTCTAGAGAGAGCATTGGCCA
AGGCCAACCCAGCCATGAGAGAGAGACAGAGAGAGGCAGGGC

---

[a]underline shows sequence of T7 promoter.
