# Supplementary material for: LINC00842 inactivates transcription co-regulator PGC-1α to promote pancreatic cancer malignancy through metabolic remodelling
Source: Nat Commun. 2021 Jun 22;12:3830. doi: 10.1038/s41467-021-23904-4 (PMC8219694; doi:10.1038/s41467-021-23904-4)
Supplement: Supplementary file 3 — Description of Additional Supplementary Files [file 41467_2021_23904_MOESM3_ESM.pdf]

Description of Additional Supplementary Files

File Name: Supplementary Data 1
Description: Prognosis-related lncRNAs in pateints with PDAC data derived from TCGA database.

File Name: Supplementary Data 2
Description: Transcription factors binding site prediction in LINC00842 promoter (JASPAR database and AnimalTFDB database, respectively)
