# Supplementary material for: LINC00842 inactivates transcription co-regulator PGC-1α to promote pancreatic cancer malignancy through metabolic remodelling
Source: Nat Commun. 2021 Jun 22;12:3830. doi: 10.1038/s41467-021-23904-4 (PMC8219694; doi:10.1038/s41467-021-23904-4)
Supplement: Supplementary file 6 — Reporting Summary [file 41467_2021_23904_MOESM6_ESM.pdf]

nature research

Corresponding author(s): Jian Zheng

Last updated by author(s): Apr 23, 2021

Reporting Summary

Nature Research wishes to improve the reproducibility of the work that we publish. This form provides structure for consistency and transparency in reporting. For further information on Nature Research policies, see our Editorial Policies and the Editorial Policy Checklist.

Statistics

For all statistical analyses, confirm that the following items are present in the figure legend, table legend, main text, or Methods section.

n/a	Confirmed	
☐	☒	The exact sample size (*n*) for each experimental group/condition, given as a discrete number and unit of measurement
☐	☒	A statement on whether measurements were taken from distinct samples or whether the same sample was measured repeatedly
☐	☒	The statistical test(s) used AND whether they are one- or two-sided<br>*Only common tests should be described solely by name; describe more complex techniques in the Methods section.*
☒	☐	A description of all covariates tested
☐	☒	A description of any assumptions or corrections, such as tests of normality and adjustment for multiple comparisons
☐	☒	A full description of the statistical parameters including central tendency (e.g. means) or other basic estimates (e.g. regression coefficient) AND variation (e.g. standard deviation) or associated estimates of uncertainty (e.g. confidence intervals)
☐	☒	For null hypothesis testing, the test statistic (e.g. *F*, *t*, *r*) with confidence intervals, effect sizes, degrees of freedom and *P* value noted<br>*Give P values as exact values whenever suitable.*
☒	☐	For Bayesian analysis, information on the choice of priors and Markov chain Monte Carlo settings
☒	☐	For hierarchical and complex designs, identification of the appropriate level for tests and full reporting of outcomes
☐	☒	Estimates of effect sizes (e.g. Cohen's *d*, Pearson's *r*), indicating how they were calculated

Our web collection on statistics for biologists contains articles on many of the points above.

Software and code

Policy information about availability of computer code

Data collection	Illumina HiSeq2500; Thermo Fisher Scientfic ultrahigh performance liquid chromatograph coupled with TSQ Quantiva mass spectrometer system (TraceFinder 3.2); AB Sciex 4000 QTrap system (ChemoView 2.0.2); Thermo Fisher Scientfic Trace 1300
Data analysis	Microsoft Excel 2019, Image J (1.50i), Photoshop CS6 (Adobe), GraphPad Prism 8.0.1 (GraphPad, La Jolla, CA, USA), SPSS software (version 22.0; IBM SPSS)

For manuscripts utilizing custom algorithms or software that are central to the research but not yet described in published literature, software must be made available to editors and reviewers. We strongly encourage code deposition in a community repository (e.g. GitHub). See the Nature Research guidelines for submitting code & software for further information.

Data

Policy information about availability of data

All manuscripts must include a data availability statement. This statement should provide the following information, where applicable:

- Accession codes, unique identifiers, or web links for publicly available datasets
- A list of figures that have associated raw data
- A description of any restrictions on data availability

The accession number for the RNA sequencing data is GSE150216 (https://www.ncbi.nlm.nih.gov/geo/query/acc.cgi?acc=GSE150216). The proteomics datasets of LINC00842 sense and antisense strand pulldown assays on the web server: http://download.omicsbio.info/files/LINC00842/. Publicly available sources: RNA sequencing data of 177 PDAC patients were retrieved from TCGA PAAD sample level 3 access (http://gdac.broadinstitute.org, version 2016_01_28; PMID: 28810144) and lncRNAs were annotated by Ensembl (PMID: 31691826); RNA array data and clinical information of PDAC patients were also downloaded from ICGC (https://dcc.icgc.org/releases/release_28/Projects/PACA-AU). The Source Data underlying Figs. 1d–h, j, l, o, 2c–f, h, 3a–l, 4a, d, e, 5a, c, e, f, 6a–d, k, m–o, 7b–d, f, g,

i–l and Supplementary Figs. 1d, e, h, 3a–e, g, 4, 6a, d, h, 7c, d, f, 8a–h, 9a–d, f, 10a, b, d, 11a–c, 12a–j, 13a–d, 14a, b are provided in Source Data files. Unprocessed gel blot of Fig. 4c, e, g, I, 5a–d, g–l, 6e–i, m, n, p–r, 7a and Supplementary Figs. 1c, g, 5b, 6b–d, e–g, i, 14e, f, 15b are provided in Supplementary Fig. 16

Field-specific reporting

Please select the one below that is the best fit for your research. If you are not sure, read the appropriate sections before making your selection.

☒ Life sciences ☐ Behavioural & social sciences ☐ Ecological, evolutionary & environmental sciences

For a reference copy of the document with all sections, see nature.com/documents/nr-reporting-summary-flat.pdf

Life sciences study design

All studies must disclose on these points even when the disclosure is negative.

Sample size	The sample size of the TCGA was determined by the number of tumor samples analyzed with RNA sequencing. Sample size and number of independent experiments are always clearly stated in the figure legend or in the Methods section. Three to more independent results were used to perform statistical analyses. If less, no statistics were performed from these samples. For our PDAC cohorts, sample size was not statistical determined before collection. The determination of sample size is based on our experience and numerous publications, which is sufficient to generate statistically significant results.
Data exclusions	No data were excluded from analysis.
Replication	Experiments in the article were reliably reproduced, replication were described in the figure legends.
Randomization	Animals with similar age and weight were randomly allocated to experimental groups. For experiments other than animals, the samples/cells were randomized into different groups prior to treatment.
Blinding	Investigators were not blinded to group allocation during data collection and/or analysis due to the complex experimental design and limited researchers.

Reporting for specific materials, systems and methods

We require information from authors about some types of materials, experimental systems and methods used in many studies. Here, indicate whether each material, system or method listed is relevant to your study. If you are not sure if a list item applies to your research, read the appropriate section before selecting a response.

Materials & experimental systems

n/a	Involved in the study	
☐	☒	Antibodies
☐	☒	Eukaryotic cell lines
☒	☐	Palaeontology and archaeology
☐	☒	Animals and other organisms
☐	☒	Human research participants
☒	☐	Clinical data
☒	☐	Dual use research of concern

Methods

n/a	Involved in the study	
☒	☐	ChIP-seq
☒	☐	Flow cytometry
☒	☐	MRI-based neuroimaging

Antibodies

Antibodies used

Rabbit anti-H2AFZ antibody (WB: dil. 1:1000, Supplier: Cell Signaling Technology, Cat.: 2718)
Rabbit anti-FBL antibody (WB: dil. 1:1000, Supplier: Cell Signaling Technology, Cat.: 2639)
Rabbit anti-HDAC1 antibody (WB: dil. 1:1000, Supplier: Cell Signaling Technology, Cat.: 34589)
Rabbit anti-acetyl-Lys antibody (WB: dil. 1:1000, Supplier: Cell Signaling Technology, Cat.: 9441)
Rabbit anti-YY1 antibody (WB: dil. 1:1000; 10 ug for ChIP, Supplier: Cell Signaling Technology, Cat.: 46395)
Rabbit anti-FASN antibody (WB: dil. 1:1000, Supplier: Cell Signaling Technology, Cat.: 3180)
Rabbit anti-GAPDH antibody (WB: dil. 1:2000, Supplier: Cell Signaling Technology, Cat.: 5174)
Rabbit anti-HIST1H2AG antibody (WB: dil. 1:2000, Supplier: Invitrogen, Cat.: PA5-24822)
Rabbit anti-HIST1H1E antibody (WB: dil. 1:2000, Supplier: Invitrogen, Cat.: PA5-31908)
Rabbit anti-HIST2H2BF antibody (WB: dil. 1:2000, Supplier: Invitrogen, Cat.: PA5-44511)
Rabbit anti-SNRPE antibody (WB: dil. 1:2000, Supplier: Invitrogen, Cat.: PA5-96342)
Rabbit anti-GCN5 antibody (WB: dil. 1:1000; 5 ug for IP, Supplier: Invitrogen, Cat.: MA5-14884)
Rabbit anti-PGC-1α antibody (WB: dil. 1:1000; 5 ug for IP and RIP, Supplier: Novus Biologicals, Cat.: NBP1-04676)
Mouse anti-SIRT1 antibody (WB: dil. 1:1000; 5 ug for IP and RIP, Supplier: Abcam, Cat.: ab110304)
Mouse anti-Flag tag antibody (WB: dil. 1:2000; 5 ug for RIP, Supplier: Sigma, Cat.: F1804)
Mouse anti-β-ACTIN antibody (WB: dil. 1:20000, Supplier: Proteintech, Cat.: 66009-1-Ig)

	Donkey anti-Rabbit IgG (H+L) Highly Cross-Adsorbed Secondary Antibody, Alexa Fluor® 488 (IF: dil. 1:500; Supplier: Invitrogen, Cat.: A-21206)
Validation	Based on the information on the user guide of antibody, when manufacturer validation was doubtful, antibody was validated by western blotting in PDAC cell lines with targeted siRNA or siControl treatment.

Eukaryotic cell lines

Policy information about cell lines

Cell line source(s)	Human PDAC cell lines PANC-1 and SW1990 and human embryonic kidney cell line 293T were purchased from the Cell Bank of Type Culture Collection of the Chinese Academy of Sciences Shanghai Institute of Biochemistry and Cell Biology. Human immortalized pancreatic duct epithelial cell line HPDE6-C7 was purchased from Biotechnology Company.
Authentication	All cell lines are commercial and authenticated by DNA fingerprinting analysis using short-tandem repeat (STR) markers.
Mycoplasma contamination	All cell lines were tested to be mycoplasma negative.
Commonly misidentified lines (See ICLAC register)	No cell lines used in this study were found in the database of commonly misidentified cell lines that is maintained by ICLAC and NCBI Biosample.

Animals and other organisms

Policy information about studies involving animals; ARRIVE guidelines recommended for reporting animal research

Laboratory animals	Female BALB/c nude mice aging 5 weeks and 4-week-old female NSG mice (F1, F2 and F3 for PDX model) purchased from the Beijing Vital River Laboratory Animal Technology were used in this study. Nude mice and NSG mice were allowed to acclimate to local conditions for 1 week and maintained under a 12 h dark/12 h light cycle with adequate food and water.
Wild animals	No wild animals were used in this study.
Field-collected samples	No field-collected samples were used in this study.
Ethics oversight	All animal experiments were approved by the Institutional Animal Care and Use Committee of Sun Yat-Sen University and performed in accordance with relevant institutional and national guidelines and regulations.

Note that full information on the approval of the study protocol must also be provided in the manuscript.

Human research participants

Policy information about studies involving human research participants

Population characteristics	Surgically removed PDAC and the corresponding adjacent normal tissue samples (n = 227) were obtained from Sun Yat-sen Memorial Hospital, Sun Yat-sen University Cancer Center (Guangzhou, China, n = 158) and Cancer Hospital, Chinese Academy of Medical Sciences (Beijing, China, n = 69) between 2005 and 2018. PDAC was diagnosed by histopathological examination. All clinicopathological information including Age, Sex, Differentiation, Lymph node metastasis, Vascular invasion, Neural invasion, TNM stage, Smoking status, Drinking status, Treatment and Clinical stage is provided in Supplementary Table 3.
Recruitment	Clinical samples were collected from Sun Yat-sen Memorial Hospital (Guangzhou, China), Sun Yat-sen University Cancer Center (Guangzhou, China) and Cancer Hospital, Chinese Academy of Medical Sciences (Beijing, China). The characteristics and clinical data of patients were acquired from medical records. All the individuals underwent pancreatectomy received no chemotherapy or radiotherapy before surgery. The bio-specimens from each individual were collected at the time of surgery. There may be selection bias, but it has little effect on the results of our study for the results of LINC00842 expression or survival analysis have been further confirmed using data obtained from TCGA and ICGC cohort.
Ethics oversight	This study was approved by the Institutional Review Board of Sun Yat-sen Memorial Hospital, Sun Yat-sen University Cancer Center and Cancer Hospital, Chinese Academy of Medical Sciences.

Note that full information on the approval of the study protocol must also be provided in the manuscript.
